# Statistical Framework for Multiresponse Analyses: Comparative Assessment of Surface Activation Strategies for 3D-Printed Carbon Black/Polylactic Acid Electrodes toward Electrochemical (Bio)Sensing Applications

**DOI:** 10.1021/acsomega.6c05769

**Published:** 2026-09-25

**Authors:** Leonan dos Santos Rodrigues, Guilherme Sales da Rocha, Franccesca Fornasier, Andresa Viana Ramos, Elton Jorge da Rocha Rodrigues, José Carlos Costa da Silva Pinto, João Victor Nicolini, Helen Conceição Ferraz

**Affiliations:** Chemical Engineering Program, Alberto Luiz Coimbra Institute for Graduate Studies and Research in Engineering (Coppe), Federal University of Rio de Janeiro, 21941-914 Rio de Janeiro, RJ, Brazil

## Abstract

Additive manufacturing using conductive polymeric filaments offers a powerful strategy for fabricating electrochemical (bio)­sensors. However, the insulating polymer matrix often limits electron transfer, demanding surface activation to expose conductive fillers and enhance electrochemical performance. Even electrodes made from bespoke filaments may demand activation. While some reagent-free treatments may require automation or careful process control to minimize operator-dependent variability, and biological treatments may involve longer processing times, chemical and electrochemical activations have provided promising results. Yet, the absence of standardized, statistically validated protocols can lead to unnecessarily complex or costly procedures, as optimal treatments are often selected based on isolated electrochemical metrics rather than on a comprehensive statistical assessment. To address this gap, the present work introduces a statistical framework for multiresponse comparison, applicable to any experimental system requiring simultaneous evaluation of multiple variables, and demonstrates its use through a case study on 3D-printed carbon black/polylactic acid (CB/PLA) electrodes fabricated via fused deposition modeling (FDM). Electrodes subjected to chemical, electrochemical, and combined activations were evaluated using the [Fe­(CN)_6_]^3–/4–^ redox probe. A multiresponse approach was employed, using a weighted performance function (*L*) to aggregate normalized electrochemical variables (anodic peak current, peak current ratio, peak-to-peak separation, and charge transfer resistance). This function was further converted into a risk-adjusted score (*L*_99%_), penalizing variability and enabling robust statistical analysis. Alkaline treatments proved the most effective activation route. The best condition combined 3.0 mol L^–1^ NaOH for 30 min with subsequent electrochemical activation in 0.1 mol L^–1^ NaOH, although the simpler chemical treatment alone offered similar performance, making it more practical for future optimization. Beyond identifying effective activation protocols for 3D-printed electrodes, this work provides a versatile statistical framework for multiresponse analyses that can serve as a practical guide for the objective comparison and optimization of diverse systems, including filament development, aging assessment, nanomaterial integration, bioreceptor immobilization, and any application involving the simultaneous evaluation of multiple variables.

## Graphical abstract

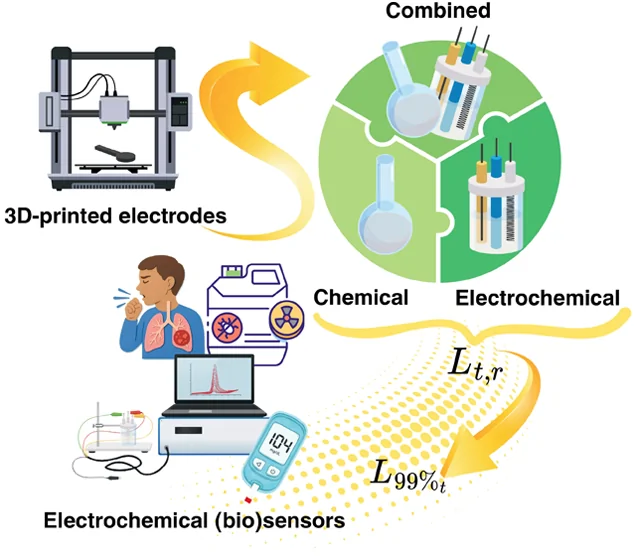

## 1 Introduction

Additive manufacturing technologies, commonly termed three-dimensional (3D) printing, have emerged as versatile and innovative tools for fabricating customized devices and prototypes.^1,2^ Their ability to enable the tailored production of laboratory equipment and analytical devices has attracted considerable attention, with applications spanning multiple sectors, including aerospace, automotive, medical, food, electronics, and energy.^3,4^

Unlike traditional subtractive manufacturing methods that involve removing material through milling or drilling,^5,6^ 3D printing builds structures layer-by-layer. This additive process substantially reduces material waste and minimizes costs associated with time, logistics, storage, and energy consumption.^5,7^ Furthermore, integrating 3D printing with plastic recycling and reuse initiatives can enhance sustainability by supporting environmental, social, and governance (ESG) criteria and circular economy principles.^8–10^

In electrochemistry, additive manufacturing holds great promise for the cost-effective, versatile fabrication of (bio)­sensors. Among the available techniques, fused deposition modeling (FDM), which employs thermoplastic filaments embedded with conductive materials, is the most widely used for producing such devices.^11,12^ These electrochemical (bio)­sensors are recognized for their high selectivity and sensitivity, combined with simplicity and affordability, which have driven their widespread application in healthcare, food safety, and environmental monitoring.^13–15^

However, a notable limitation in the performance of 3D-printed electrodes arises from the inherently insulating nature of the polymer matrix within the filaments, which requires surface post-treatments to expose conductive material and enhance electrical conductivity.^2,16^ Although the development of bespoke filaments enables control over conductive filler composition (often improving electrochemical performance and, in some cases, eliminating the need for activation^17^), surface activation is still often required because the electrochemical performance of 3D-printed electrodes remains dependent on factors such as filament formulation, filler dispersion quality, processing parameters, and printing conditions.^8^ To address this challenge, various post-treatment strategies have been investigated, including chemical and photochemical procedures,^18–20^ electrochemical activation,^21–23^ enzymatic treatment,^24,25^ and reagent-free methods, such as mechanical polishing,^26,27^ thermal activation,^28^ spark discharge,^29^ and laser/plasma treatments.^30–32^

Biological treatments tend to be more time-consuming because they rely on slow enzymatic activity, and the effectiveness of reagent-free strategies may be compromised when automation is not employed or when process variables are not carefully controlled, leading to inconsistent outcomes and poor electrode-to-electrode reproducibility due to their sensitivity to operational conditions.^33^ Conversely, chemical treatments, alone or combined with electrochemical processes, have been widely investigated as relatively simple and cost-effective strategies for surface modification.^16,34,35^

Despite the breadth of approaches, consensus on the optimal conditions, including reagent concentration, treatment duration, and electrochemical protocols, remains elusive. Furthermore, investigations often select optimal treatments based solely on electrochemical metrics without employing statistical analyses for validation and comparison, potentially leading to a preference for more complex and costly procedures without significant performance advantages.^33^

In light of these challenges, the present study addresses this limitation by proposing a statistical framework to evaluate the efficacy of chemical, electrochemical, and combined surface post-treatments applied to 3D-printed electrodes fabricated from the commercial ProtoPasta filament, a composite of carbon black (CB) in a polylactic acid (PLA) matrix, via fused deposition modeling (FDM). Beyond identifying more efficient, sustainable, and reproducible activation protocols for the production of electrochemical (bio)­sensors, this work aims to provide a methodological contribution that can be readily extended to other systems requiring the simultaneous assessment of multiple variables, thereby offering a practical tool to guide statistically robust decision-making across different fields.

## 2 Materials and Methods

### 2.1 Chemicals

All chemicals used in this study were of analytical grade and used without further purification. Sulfuric acid (95–97%, H_2_SO_4_), potassium chloride (≥99%, KCl), potassium ferricyanide (≥99%, K_3_[Fe­(CN)_6_]), potassium dihydrogen phosphate (≥99.5%, KH_2_PO_4_), and disodium hydrogen phosphate (≥99%, Na_2_HPO_4_) were purchased from Vetec (Brazil). Potassium ferrocyanide trihydrate (98.5–102.0%, K_4_[Fe­(CN)_6_]·3H_2_O) and N,N-dimethylformamide (≥99.8%, DMF – (CH_3_)­2NC­(O)­H) were obtained from Sigma-Aldrich (USA). Sodium hydroxide (≥97%, NaOH) was purchased from Neon (Brazil). Sodium chloride (≥99%, NaCl) was supplied by Isofar (Brazil). Polylactic acid (PLA) pellets Ingeo 4043D (≥98%) were acquired from NatureWorks (USA), and the conductive filament (CB/PLA) was obtained from Protopasta, Protoplant (USA). All experiments used ultrapure water (resistivity: 18.2 MΩ·cm at 25 °C; conductivity: 0.056 μS cm^–1^ at 25 °C), obtained from a Direct-Q Millipore/Merck purification system (KGaA, Molsheim, France).

### 2.2 Fabrication and Activation of 3D-Printed Electrodes

Pendulum-shaped electrodes (52.5 × 5 × 2 mm) were fabricated in triplicate with a fused deposition modeling (FDM) 3D printer (Ender-3S1 Plus, Creality, China) using the CB/PLA filament (Figure S1 of the Supporting Information). The 3D design was created in FreeCAD, processed in Simplify3D, and printed on a glass bed. The printing parameters were as follows: an extrusion temperature of 210 °C, a bed temperature of 60 °C, a printing speed of 30 mm s^–1^, a layer height of 0.2 mm, and a 100% infill density with a grid pattern. Additional settings included a first layer height of 100%, a contour overlap of 15%, two perimeter shells, and an extrusion multiplier of 1.1. After fabrication, the electrodes were stored at room temperature until further use.

On the basis of the scientific literature, the activation procedures included chemical, electrochemical, and combined treatments.^22,33,35^ Prior to the comparative evaluation, preliminary screening experiments were conducted to define the activation conditions employed throughout the study. For chemical activation, an initial assessment was performed to determine whether ultrasonic assistance improved the activation process (Figure S2 of the Supporting Information). Because no enhancement in the electrochemical performance was observed, subsequent chemical treatments were conducted without ultrasound. The chemical protocols were selected to represent three widely employed activation approaches for CB/PLA electrodes: acidic activation (H_2_SO_4_), alkaline treatment (NaOH), and solvent activation (DMF), all of which have been extensively reported for promoting the partial removal of the insulating PLA matrix and increasing the exposure of conductive carbonaceous domains.^22,33,35^

Similarly, a preliminary electrochemical screening was carried out to identify the most suitable electrochemical activation protocol. Different potential windows and electrochemical procedures were evaluated (Figure S3 of the Supporting Information), and five cyclic voltammetric cycles between +2.5 V and −2.5 V (100 mV s^–1^) were selected based on their superior electrochemical performance. Electrochemical activation was investigated in either 0.1 mol L^–1^ NaOH or 0.1 mol L^–1^ phosphate-buffered saline (PBS, pH 7.0), as both electrolytes have been widely employed for the activation of PLA-based conductive composite electrodes.^22,33,35^ A schematic representation of the chemical, electrochemical, and combined activation procedures applied to CB/PLA electrodes is illustrated in Scheme 1. The electrochemical cell was filled to capacity (50 mL) to ensure complete immersion of the electrode, with only the end of the stem exposed for consistent electrical contact.

**Scheme 1:**
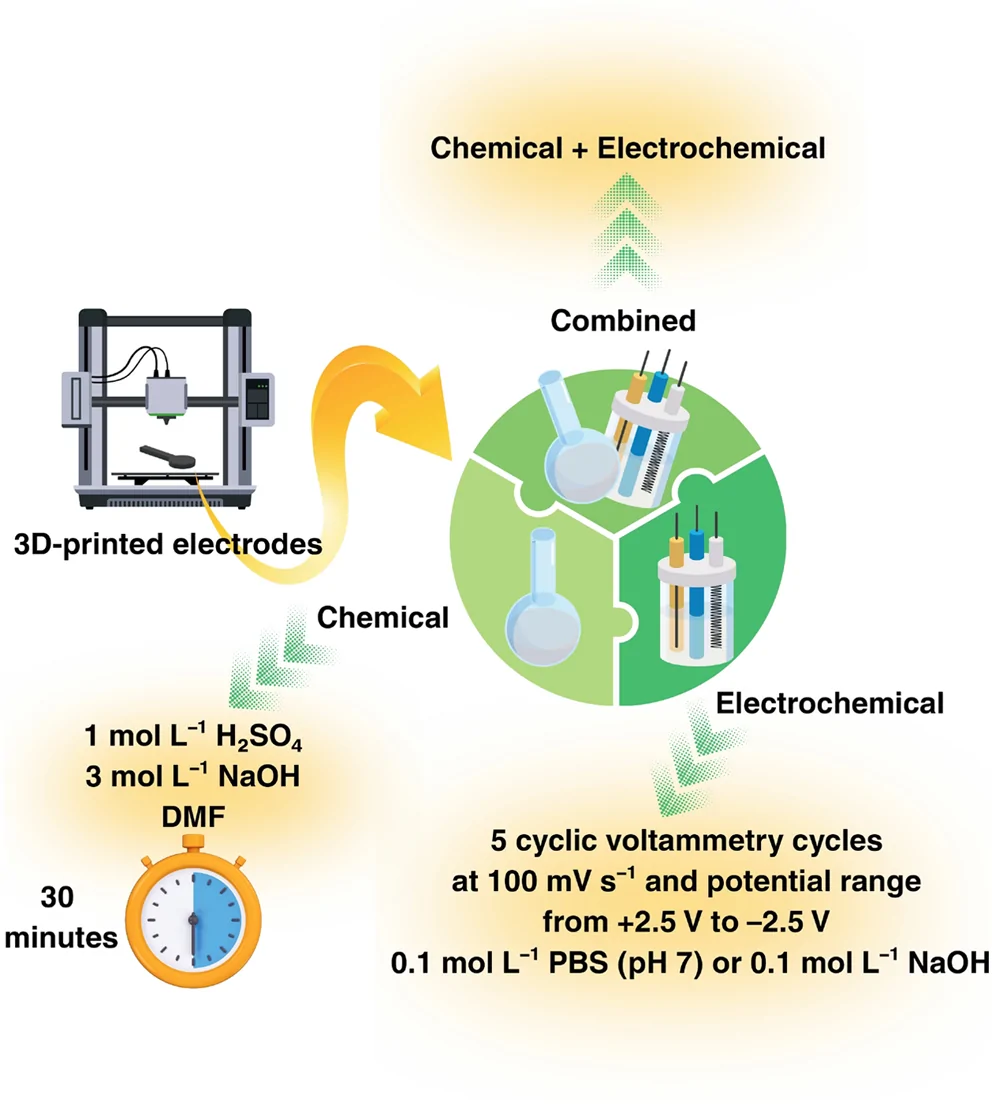
Schematic Representation of the Activation Procedures Applied to 3D-Printed CB/PLA Electrodes

After each treatment, the electrodes were rinsed with ultrapure water for 30 s. Electrodes subjected to a single treatment were then dried in an oven at 60 °C for one hour. For the combined treatments, chemical activation was followed by electrochemical treatment, as this sequence has been reported to be more effective than the reverse order.^36^ In this case, rinsing was performed after each step, while oven drying was carried out only once, after completion of the combined procedures, under the same conditions described above. This temperature was chosen to efficiently remove residual moisture remaining after the activation and rinsing steps while minimizing the risk of thermal deformation of the PLA-based printed electrodes, in agreement with drying conditions commonly employed for 3D-printed PLA-based electrodes in the literature.^35,37,38^ After drying, all electrodes were stored at ambient temperature until use.

A specific nomenclature was adopted to identify the treatments. Electrodes subjected exclusively to chemical activation were designated by the reagent used (DMF, NaOH, or H_2_SO_4_). Those undergoing only electrochemical activation were labeled as EC_NaOH_ or EC_PBS_. For the combined treatments, the nomenclature reflects the sequence of applied treatments, with the chemical treatment followed by the electrochemical activation, separated by a hyphen (e.g., DMF-EC_NaOH_).

### 2.3 Electrochemical Measurements

Electrochemical characterization of both treated and untreated electrodes was performed using cyclic voltammetry (CV) and electrochemical impedance spectroscopy (EIS). A potentiostat (EmStat4S, PalmSens, Netherlands) controlled by PSTrace 5.10 software was employed. A silver/silver chloride reference electrode (Ag|AgCl, 3 mol L^–1^ KCl) and a platinum wire counter electrode were used in all experiments. All measurements were carried out in a 50 mL electrochemical cell containing 40 mL of an equimolar 1 mmol L^–1^ solution of [Fe­(CN)_6_]^3–/4–^ in 0.1 mol L^–1^ KCl. The working, reference, and counter electrodes were immersed in the electrolyte solution, with only the circular sensing region of the 3D-printed working electrode exposed to the solution and used for electrochemical measurements. All electrolyte solutions were thoroughly purged with nitrogen gas (N_2_) for 10 min prior to electrochemical measurements to remove dissolved oxygen.

CV was conducted over a potential window from −1.2 to +1.5 V at ten different scan rates (5–200 mV s^–1^). Data from the third scan cycle were used for all analyses. The electroactive area (*A*_*e*_) of the electrodes for each treatment was estimated via nonlinear regression using the Randles–Ševčík equation (eq ([Disp-formula eq1])),^39,40^ implemented in Statistica software (TIBCO Software Inc., version 14). For each electrode, the data set consisted of the anodic peak current (*I*_*pa*_) values measured at all scan rates.

1 $$I_{\mathrm{pa}}=2.69\times 10^{5}n^{3/2}A_{e}\sqrt{D}C\sqrt{v}$$

where *I*_*pa*_ is the anodic peak current (A), *n* is the number of transferred electrons, *A*_*e*_ is the electroactive area (cm^2^), *D* is the diffusion coefficient of 1:1 mmol L^–1^ Fe­(CN)_6_^3–^/^4–^ in 0.1 mol L^–1^ KCl with a value of 6.7 × 10^–6^ cm^2^ s^–1^,^41^ *v* is the scan rate (V s^–1^), and *C* is the concentration (1 × 10^–6^ mol cm^–3^).

The homogeneity of variances across different scan rates was assessed for each surface treatment using the well-known Levene’s test.^42^ For treatments exhibiting homogeneous variances (*p* > 0.05), *A*_*e*_ was estimated using the ordinary least-squares (OLS) method, which minimizes the sum of squared residuals. However, when Levene’s test indicated heteroscedasticity (*p* ≤ 0.05), the weighted least-squares (WLS) method was employed. In this approach, the weighting factor applied to the data points at a given scan rate was the inverse of the variance (σ_*I*_*pa*__^2^) calculated from the three replicate measurements at that specific rate. This procedure assigns less weight to data from scan rates with higher variability among replicates, thus yielding a more accurate estimation of *A*_*e*_.

The heterogeneous electron transfer rate constant (*k* ^0^) was calculated using the Klingler–Kochi method (eq 2),^43,44^ based on the arithmetic mean of the values obtained across all 10 scan rates for each electrode. The charge transfer coefficient (α) was calculated with eq 3 ^45^

2 $$k^{0}=2.18\sqrt{\frac{\alpha \mathrm{DnvF}}{\mathrm{RT}}}e^{-\left(\frac{\alpha^{2}\mathrm{nF}}{\mathrm{RT}}\right)\Delta E_{p}}$$

3 |Ep−Ep/2|=1.857RTα=47.7mVα(at25C°)

where *k* ^0^ is the heterogeneous electron transfer rate constant (cm s^–1^); *D* is the diffusion coefficient of the electroactive species (cm^2^ s^–1^); *n* is the number of electrons transferred; ν is the scan rate (V s^–1^); Δ*E*_*p*_ is the peak-to-peak potential separation (V); *R* is the universal gas constant (8.314 J mol^–1^ K^–1^); *T* is the temperature (298.15 K); *E*_*p*_ and *E*_*p*/2_ are the peak and half-peak potentials (V), respectively; α is the charge transfer coefficient; and *F* is the Faraday constant (96,485 C mol^–1^).

The plots of the anodic (*I*_*pa*_) and cathodic (*I*_*pc*_) peak currents versus the square root of the scan rate (√*v*), obtained from CV data, were analyzed to investigate the mass-transport mechanism. High coefficients of determination (*R* ^2^) close to unity indicate that the electrochemical process is primarily controlled by diffusion, as predicted by eq 1. On the other hand, noticeable deviations from *R* ^2^ from unity suggest that the electrochemical process is not purely diffusion-controlled but is also influenced by surface adsorption phenomena at the electrode interface.^46^

EIS measurements were performed over a frequency range from 100 kHz to 0.1 Hz, with a sinusoidal alternating current (AC) perturbation of 10 mV amplitude under open-circuit potential (OCP) conditions. The impedance data were fitted to equivalent circuit models, as shown in Figure S4 of the Supporting Information.

### 2.4 Statistical Analysis for Selection of Promising Treatments

The electrochemical performance of 3D-printed electrodes subjected to different surface treatments was compared using CV and EIS in the presence of the [Fe­(CN)_6_]^3–/4–^ redox probe via two distinct methodologies. Both approaches followed an initial step of variable normalization. The variables *I*_*pa*_, the ratio |*I_pa_*/*I_pc_* |, and Δ*E*_*p*_, obtained from CV measurements, and the charge transfer resistance (*R*_*ct*_), determined from EIS experiments, were normalized between 0 and 1 across all treatments for each scan rate, ensuring that a value of 1 consistently represented the best performance among all treatments, according to eqs 4, 5, or 6.

4 $$X_{t,v,r,j}^{\mathrm{norm}}=\frac{X_{t,v,r,j}-X_{v,j}^{\min}}{X_{v,j}^{\max}-X_{v,j}^{\min}},\mathrm{if}\;\mathrm{higher}\;\mathrm{values}\;\mathrm{are}\;\mathrm{desirable}\;\left(I_{\mathrm{pa}}\right)$$

5 $$X_{t,v,r,j}^{\mathrm{norm}}=1-\frac{X_{t,v,r,j}-X_{v,j}^{\min}}{X_{v,j}^{\max}-X_{v,j}^{\min}},\mathrm{if}\;\mathrm{lower}\;\mathrm{values}\;\mathrm{are}\;\mathrm{better}\;\left(\Delta E_{p},R_{\mathrm{ct}}\right)$$

6 $$X_{t,v,r,j}^{\mathrm{norm}}=1-\frac{{|X}_{t,v,r,j}-1|}{\max\left(|X_{v,j}-1|\right)},\mathrm{if}\;\mathrm{the}\;\mathrm{optimum}\;\mathrm{is}\;1\;\left(|I_{\mathrm{pa}}/I_{\mathrm{pc}}|\right)$$

where *X* ^*norm*^ represents the normalized variable, *t* is the surface treatment, *v* is the scan rate, *r* is the replicate, and *j* is the electrochemical variable.

In the first approach, an individual performance score (*L*_*t*,*v*,*r*_) was computed for each surface treatment, scan rate, and replicate by combining the normalized variables into a single metric (eq ([Disp-formula eq7])). Equal weights (*w*_1_ = *w*_2_ = *w*_3_ = *w*_4_ = 0.25) were assigned to each electrochemical variable. The *R*_*ct*_ value obtained for each electrode was used identically across the ten scan rates, corresponding to that same electrode.

7 $$L_{t,v,r}=w_{1}.{I_{\mathrm{pa}}}_{t,v,r}^{\mathrm{norm}}+w_{2}.{I_{\mathrm{pa}}/I_{\mathrm{pc}}}_{t,v,r}^{\mathrm{norm}}+w_{3}.{\Delta E_{p}}_{t,v,r}^{\mathrm{norm}}+w_{4}.{R_{\mathrm{ct}}}_{t,v,r}^{\mathrm{norm}}$$

As suggested by Pinto,^47^ a risk-adjusted score (*L*_99%*t*,*v*_) was then calculated for each treatment at each scan rate to account for variability among replicates, according to eq 8.

8 $${L_{99\%}}_{t,v}=\overset{®}{L_{t,v}\;}-k.\sigma_{L_{t,v}}$$

where $\overset{®}{L_{t,v}\;}$ is the mean score of a treatment at a scan rate, σ_*L*_*t*,*v*__ is its standard deviation, and *k* = 2.35 corresponds to a 99% confidence level.

The function *L* employed in this study is conceptually analogous to a desirability function,^48^ but extended to include a statistical robustness term (−*k*.σ_*L*_), penalizing variability across replicates and ensuring more reliable optimization outcomes.^47^

In the second approach, the overall performance of each replicate for a given surface treatment (*L*_*t*,*r*_) was obtained as the weighted sum of the normalized contributions of each electrochemical variable across all scan rates, to which the contribution of *R*_*ct*_, not obtained from CV, was then added. Equal total weights (0.25) were assigned to the four-performance metrics (*I*_*pa*_, |*I_pa_*/*I_pc_* |, Δ*E*_*p*_, and *R*_*ct*_), leading to individual weights of *w*_1_ = *w*_2_ = *w*_3_ = 0.025 per variable per scan rate for the CV variables, and *w*_4_ = 0.25 for *R*_*ct*_. The total performance function was therefore defined by eq 9

9 $$L_{t,r}=\sum_{v=1}^{10}\left(w_{1}{I_{\mathrm{pa}}}_{t,v,r}^{\mathrm{norm}}+{{w_{2}|I}_{\mathrm{pa}}/I_{\mathrm{pc}}|}_{t,v,r}^{\mathrm{norm}}+{w_{3}\Delta E_{p}}_{t,v,r}^{\mathrm{norm}}\right)+w_{4}{R_{\mathrm{ct}}}_{t,r}^{\mathrm{norm}}$$

For each treatment *t*, *L*_99%*t*_ was defined as the mean of *L* over the replicates *r*, while the standard deviation (σ_*L*_*t*__) was used to compute the risk-adjusted performance according to eq 10, with *k* = 2.35 corresponding to a 99% confidence level.

10 $${L_{99\%}}_{t}=\overset{®}{L_{t}\;}-k.\sigma_{L_{t}}$$

The selection of the value *k* = 2.35 in eqs 8 and 10 establishes a conservative lower bound for the performance score at the 99% confidence level. This approach is based on the assumption that the performance scores (*L* and *L*_99%_) follow a normal distribution across experimental replicates. Although the small sample size (*n* = 3 replicates per treatment) means the true nature of the distribution cannot be reliably determined, this assumption represents a common and practical approximation in engineering analysis, supported by the general principles of the Central Limit Theorem for aggregated measures.^49^

In statistical terms, for a normally distributed variable, the theoretical *z*-value for a 99% confidence level under a normal distribution is 2.326.^50^ However, in practical engineering applications, particularly when dealing with limited replicates, it is common to use a slightly more conservative value to account for potential deviations from normality and to ensure robustness against outliers.^51^

Thus, the value *k* = 2.35 provides a practical and conservative estimate for the 99% lower confidence bound, effectively ensuring that the reported *L*_99%_ represents a performance level that the treatment is expected to exceed with a 99% confidence. This approach aligns with the risk-aversion principle advocated by Pinto,^47^ where economic value is assigned to uncertainties to support robust decision-making.

Levene’s test was performed to verify the homogeneity of variances across the different scan rates and treatments for both *L* and *L*_99%_.

### 2.5 Electrical Resistance Measurements

Electrical resistance tests (in triplicate) were carried out using linear sweep voltammetry (LSV) in the potential range of −1.0 to +1.0 V at a scan rate of 50 mV s^–1^. Electrical resistance was obtained by linear regression using Statistica software (TIBCO Software Inc., version 14), following Ohm’s law. These measurements served as an indirect approach to assess the effect of treatments on the conductivity of the electrodes. Since electrical resistance (*R*, Ω) is directly related to resistivity (ρ, Ω·m) and inversely related to conductivity (σ, S m^–1^, where *S* = Ω^–1^),^52^ a reduction in resistance for electrodes with similar length (*L*, m) and cross-sectional area (*A*, m^2^) indicates improved conductivity.

Welch’s analysis of variance (Welch’s ANOVA), a version of ANOVA that does not assume homogeneity of variances, was applied to determine whether significant differences existed between group means. Games-Howell post-hoc test was then employed to identify which specific treatments differed from each other, as it is suitable for data with unequal variances.^53^

### 2.6 Electrode Characterization

Contact angle measurements were performed in triplicate on the filament as well as on treated and untreated electrodes using the sessile drop method. Measurements were carried out with a goniometer (Dataphysics OCA 15, Germany) equipped with SCA20 software, employing a 3 μL droplet of ultrapure water. The final contact angle was calculated as the average of the left and right angles of the droplet. ANOVA and Tukey’s post hoc test were used to assess statistical differences between treatments.^53^

Scanning electron microscopy with energy-dispersive X-ray spectroscopy (SEM-EDS) analysis was conducted using a VEGA3 microscope (TESCAN, Czech). Samples were fixed on aluminum stubs using carbon tape and sputter-coated with platinum. SEM images were obtained under a 10.0 keV accelerating voltage, and EDS spectra were acquired at 20.0 keV and 80× magnification.

Attenuated total reflection Fourier transform infrared (ATR-FTIR) was performed using a VERTEX 70 spectrometer (BRUKER, Germany). Due to surface degradation from the treatments, it was expected that more intense carbon black characteristic bands would be observed. Spectra were recorded from 4000 to 400 cm^–1^ at a resolution of 4 cm^–1^. Raman spectroscopy was performed using a Raman microscope (DRX, Thermo Scientific, USA) equipped with a 532 nm laser, measuring across a Raman shift range of 200 to 3500 cm^–1^ at a spectral resolution of 2 cm^–1^.

Thermogravimetric analysis (TGA) was performed using a Pyris 1 system (PerkinElmer, USA) with heating from 50 to 600 °C at 2 °C min^–1^ under an inert atmosphere. For comparison, a pure PLA filament produced by extrusion (Filmaq3D CV, Brazil) was analyzed alongside the filament and electrodes. Dynamic mechanical analysis (DMA) was conducted using a DMA 8000 instrument (PerkinElmer, USA), performed from room temperature to 100 °C at a heating rate of 2 °C min^–1^, also including the pure PLA reference.

To facilitate the interpretation of the experimental procedures, particularly for readers less familiar with the techniques and statistical methods employed, Table 1 summarizes the electrochemical techniques, physicochemical characterization methods, and statistical analyses used throughout this study. For each method, a brief description, the corresponding response variables, and their specific application in the present work are provided.

**Table 1:** Summary of the Electrochemical Techniques, Physicochemical Characterization Methods, and Statistical Analyses Employed in This Study, Including a Brief Description of Each Method or Test, the Corresponding Response Variables, and Their Specific Applications

technique/test	brief description	application in this study
Cyclic voltammetry (CV)	An electrochemical technique widely used to characterize electrode materials by monitoring the current response during cyclic potential sweeps. Key data extracted from the voltammograms include the anodic and cathodic peak currents (*I*_*pa*_ and *I*_*pc*_, respectively), the peak current ratio (|*I_pa_*/*I_pc_* |), and peak-to-peak separation (Δ*E*_*p*_), which provide information on electron-transfer kinetics, electroactive surface area, reversibility, and mass-transport processes.^45^	Used to perform electrochemical activation, evaluate the electrochemical performance of the electrodes toward the [Fe(CN)_6_]^3–/4–^ couple, and calculate the apparent electroactive area (*A*_*e*_) and the apparent heterogeneous electron transfer rate constant (*k* ^0^). *I*_*pa*_, |*I_pa_*/*I_pc_* |, and Δ*E*_*p*_ were used as variables in the statistical framework proposed in this study to compare the effect of the different surface treatments on electrochemical performance.
Electrochemical impedance spectroscopy (EIS).	A technique that applies a small alternating potential perturbation over a range of frequencies and measures the resulting current response, allowing the deconvolution of processes such as charge transfer resistance, solution resistance, and double-layer capacitance.^45^	Used to quantify the charge transfer resistance (*R*_*ct*_) at the electrode/electrolyte interface under different surface treatments and as an input variable in the statistical framework proposed in this study.
Linear sweep voltammetry (LSV)	A voltammetric technique in which the potential is swept in a single direction (without a reverse scan), commonly used to investigate oxidation or reduction processes, particularly irreversible electrochemical reactions.^45^	Applied for complementary characterization through estimation of the apparent electrical resistance based on the current–potential relationship according to Ohm’s law.
Chronoamperometry (CA)	A technique in which the electrode potential is stepped to a fixed value and the transient current response is monitored over time, often reflecting mass-transport and interfacial electrochemical processes.^45^	Used as an electrochemical activation protocol during the preliminary screening of activation conditions.
Levene’s test	A statistical test used to assess the homogeneity of variances across two or more groups by comparing the variability of observations within each group. Based on the predefined significance level (α = 0.05 in this study), *p* < α indicates heterogeneity of variances.^42^	Used to check whether variances were homogeneous across the experimental groups, determining whether classical ANOVA or Welch’s ANOVA was more appropriate.
Shapiro–Wilk test	A statistical test used to assess whether a sample follows a normal distribution. Based on the predefined significance level (α = 0.05 in this study), *p* < α indicates evidence against the assumption of normality.^54^	Used to assess the assumption of normality prior to selecting the parametric statistical test (e.g., ANOVA or Welch’s ANOVA).
Analysis of variance (ANOVA)	A statistical method used to test whether at least one of three or more group means differs from the others, by comparing the ratio of between-group to within-group variability (*F*-statistic). A *p*-value below the predefined significance level (α = 0.05 in this study) indicates that at least one group mean differs significantly.^53,55^	Applied when data met the assumptions of normality and homogeneity of variances; otherwise, Welch’s ANOVA was used.
Welch’s analysis of variance (Welch’s ANOVA)	A version of ANOVA that does not assume equal variances across groups, testing whether at least one group mean differs from the others by adjusting the degrees of freedom to account for heteroscedasticity.^53^	Applied when Levene’s test indicated heterogeneity of variances among the experimental groups.
Tukey’s test	A post hoc multiple-comparison test applied after a significant ANOVA to identify which pairs of group means differ while controlling the family-wise probability of Type I errors (false-positive results) under the assumption of homogeneous variances. A *p* < α (α = 0.05 in this study) indicates a significant difference between the compared groups.^55^	Applied to perform pairwise comparisons of contact angle values after a significant ANOVA when the assumption of homogeneity of variances was satisfied.
Games-Howell post hoc test	A post hoc multiple-comparison test applied after a significant Welch’s ANOVA to identify which pairs of group means differ while controlling the family-wise probability of Type I errors (false-positive results) without assuming equal variances or equal sample sizes. A *p* < α (α = 0.05 in this study) indicates a significant difference between the compared groups. It is the appropriate counterpart to Welch’s ANOVA.^53^	Used for pairwise comparisons of the electrical resistance among treatments when Welch’s ANOVA indicated a significant difference and the assumption of homogeneity of variances was violated.
Contact angle measurements (sessile drop method)	A technique that measures the angle formed between a liquid droplet and a solid surface to assess surface wettability, providing indirect information about changes in surface energy and hydrophilicity/hydrophobicity.^56^	Used to evaluate changes in the surface wettability of the electrodes after the different activation treatments and indirectly infer modifications in surface composition, including possible exposure of the carbon black phase.
Scanning electron microscopy with energy-dispersive X-ray spectroscopy (SEM-EDS)	SEM produces high-resolution images of sample topography by scanning the surface with an electron beam; EDS, coupled to the SEM, identifies elemental composition from the characteristic X-rays emitted.^57^	Used to characterize the surface morphology and elemental composition of the filament and electrodes before and after the activation treatments.
Attenuated total reflection Fourier transform infrared (ATR-FTIR)	A vibrational spectroscopic technique in which infrared light is directed through a high-refractive-index crystal in close contact with the sample. Total internal reflection generates an evanescent wave that penetrates into the sample. The resulting spectrum exhibits characteristic bands corresponding to specific functional groups based on their vibrational modes.^56^	Used to evaluate chemical modifications induced by the different surface treatments, including the formation or removal of surface functional groups associated with the activation process.
Raman spectroscopy	A vibrational spectroscopic technique based on inelastic light scattering that provides structural information on carbon materials through characteristic Raman bands (e.g., *D* and *G*). Parameters such as the *I*_*D*_/*I*_*G*_ intensity ratio and the full width at half-maximum (FWHM) of the Raman bands provide information on the degree of structural disorder, defect density, and graphitic ordering.^58,59^	Used to evaluate structural changes in the carbon black phase after the different surface treatments through analysis of the *I*_*D*_/*I*_*G*_ ratio and the FWHM of the *D* and *G* bands.
Thermogravimetric analysis (TGA)	A thermal analysis technique that monitors the mass of a sample as a function of temperature (or time under controlled temperature), enabling the identification of mass-loss events, thermal stability, and the relative composition of material constituents.^60^	Used to determine the carbon black fraction in the CB/PLA filament and to investigate the thermal stability of the samples.
Dynamic mechanical analysis (DMA)	A technique in which an oscillatory mechanical load is applied to a material while the resulting deformation is measured, enabling determination of viscoelastic properties such as the storage modulus (*E*′), loss modulus (*E*″), damping factor (tan δ), and glass transition temperature (*T*_g_).^60^	Used to evaluate the viscoelastic behavior and mechanical properties of the pure PLA filament, the CB/PLA filament, and the 3D-printed electrodes.
Multiresponse statistical framework (this work)	A multicriteria approach that integrates multiple normalized response variables into a single weighted performance function (*L*), avoiding reliance on individual variables that may differ in magnitude and reproducibility, followed by calculation of a risk-adjusted score (*L*_99%_), that penalizes variability among replicates. This approach enables simultaneous evaluation of multiple performance metrics while improving the robustness and reliability of comparisons.	Developed to compare the overall electrochemical performance of electrodes subjected to different surface activation treatments by integrating *I*_*pa*_, |*I_pa_*/*I_pc_* |, Δ*E*_*p*_, and *R*_*ct*_ into a single performance metric (*L*_99%_). This approach enables objective ranking of treatments by identifying the best-performing and most reproducible surface activation protocols.

## 3 Results and Discussion

### 3.1 Electrochemical Performance of the 3D-Printed Electrodes

A preliminary screening was performed to establish the operating conditions for the chemical and electrochemical treatments, following procedures adapted from the literature.^33^ For the chemical treatment, the influence of the ultrasonic bath (7Lab, Brazil) was evaluated under controlled conditions (3.8 L total capacity, 40 kHz working frequency, heater turned off). The electrodes (*n* = 3) were either left untreated or immersed for 30 min at room temperature in ultrapure water or 3 mol L^–1^ NaOH solution, with and without ultrasound. The results (Figure S2 of the Supporting Information) demonstrated that the ultrasonic bath did not enhance the electrochemical performance of the electrodes compared to the treatment conducted without ultrasound. Electrodes immersed in 3 mol L^–1^ NaOH for 30 min without ultrasound exhibited higher redox peak currents and improved reversibility for the [Fe­(CN)_6_]^3–^/^4–^ redox couple. This improvement may be related to a more uniform diffusion of hydroxide ions across the electrode surface in the absence of acoustic perturbations, favoring the hydrolysis of ester bonds within the PLA matrix and greater exposure of the conductive carbon black domains. The use of ultrasound induced the formation and implosion of microbubbles (acoustic cavitation), generating localized turbulence and high-energy microcurrents^61^ that may have disturbed the uniform diffusion of hydroxide ions at the CB/PLA interface. While this phenomenon is generally reported to enhance mixing and mass transfer, it may have reduced the effective contact time between hydroxide ions and the polymeric ester domains, resulting in a less uniform and incomplete saponification process compared to the static immersion condition. Therefore, in this work, the chemical treatments were performed without the use of an ultrasonic bath to ensure a more controlled and homogeneous surface activation. The exact mechanisms underlying this lack of improvement by the ultrasonic bath would require complementary surface characterization, which was beyond the scope of the present screening experiment.

Regarding electrochemical activation, several protocols were investigated in 0.1 mol L^–1^ NaOH solution:

- Application of +1.8 V for 15 min, followed by one CV cycle from 0.0 to −1.8 V at 50 mV s^–1^;
- Chronoamperometry (CA) at +1.4 V and −1.0 V, respectively, for 200 s each;
- Application of +2.5 V for 10 min, followed by one CV cycle from 0.0 to −2.5 V at 50 mV s^–1^;
- CA at +2.5 V and −2.5 V, respectively, for 5 min each;
- Five consecutive CV cycles from −2.5 V to +2.5 V at 100 mV s^–1^;
- Five consecutive CV cycles from +2.5 V to −2.5 V at 100 mV s^–1^.

Among all tested conditions, the treatments involving five CV cycles between −2.5 V and +2.5 V (or vice versa) at 50 mV s^–1^ showed the best electrochemical performance (Figure S3 of the Supporting Information). Electrodes treated under these conditions exhibited enhanced redox currents and improved reversibility toward the [Fe­(CN)_6_]^3–^/^4–^ redox probe. The combination of wide potential windows and multiple cycles likely favored the removal of residual PLA and the formation of electroactive surface sites, thereby increasing the electroactive area and improving charge-transfer kinetics. Similar results were observed between the two scan directions. However, considering potential future applications involving bioreceptor immobilization, the CV treatment that ends at a positive potential (+2.5 V) was chosen as the electrochemical activation protocol. Finishing the activation under positive potentials is expected to generate oxygen-containing functional groups, such as hydroxyl, carbonyl, and carboxyl species, on the electrode surface,^62^ which facilitate covalent biomolecule immobilization. A schematic representation of the preliminary screening protocols for chemical activation (with and without ultrasound assistance) and electrochemical activation (various potential and time protocols in 0.1 mol L^–1^ NaOH) is illustrated in Scheme 2.

**Scheme 2:**
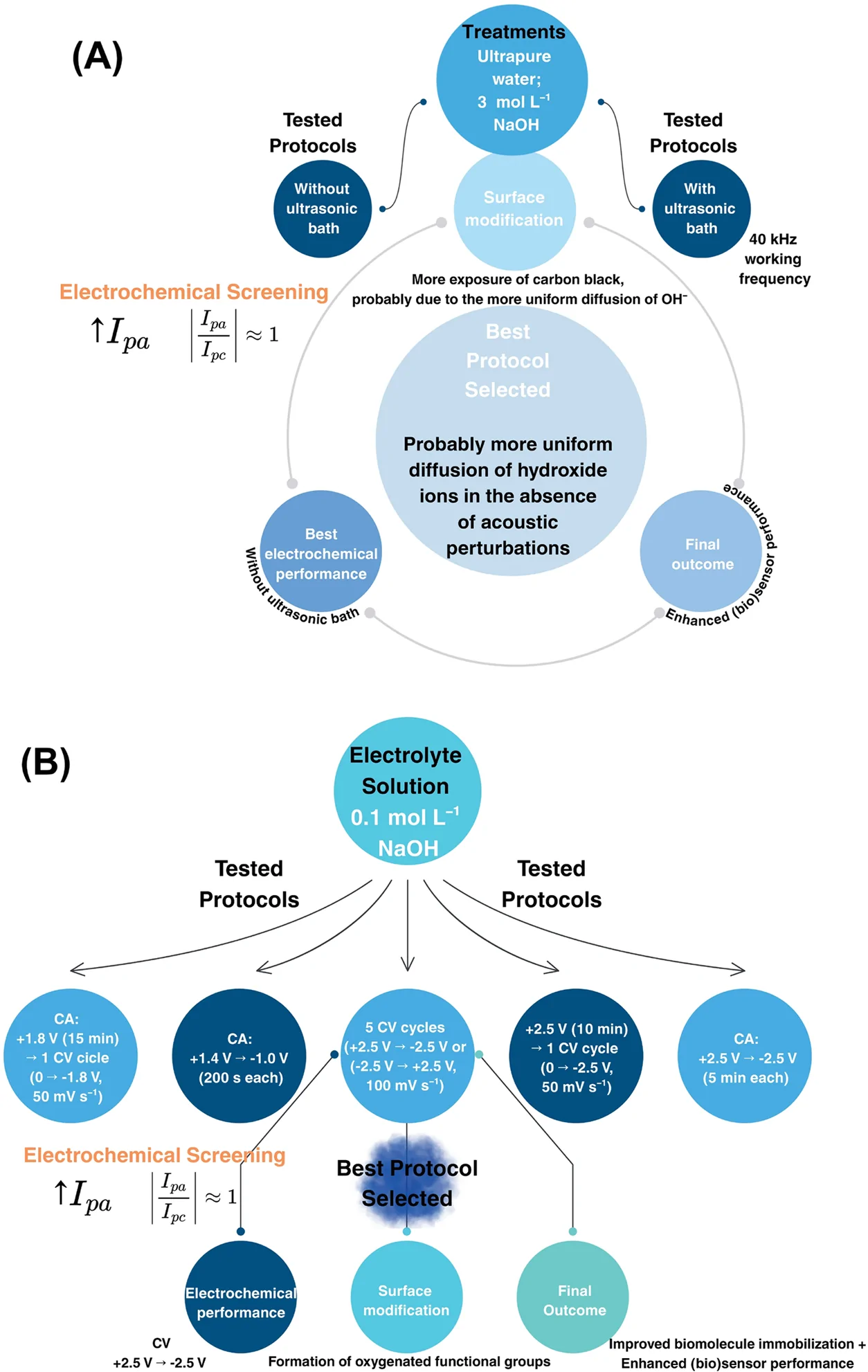
Schematic Representation of the Preliminary Screening Protocols for Chemical and Electrochemical Activation of CB/PLA Electrodes^a^

a (A) Chemical activation tests comparing immersion in ultrapure water and 3 mol L^–1^ NaOH for 30 min at room temperature with and without ultrasound assistance (40 kHz working frequency). (B) Overview of the electrochemical activation protocols evaluated in 0.1 mol L^–1^ NaOH.

After the activation procedures described in the experimental section, the 3D-printed electrodes were electrochemically characterized. Representative CVs for each scan rate and treatment are presented in Figures S5–S10 of the Supporting Information, along with the corresponding plots of *I*_*p*_ versus *v* ^1/2^, including standard deviations, and Nyquist plots. The voltammetric profiles recorded during the electrochemical activation screening (Figure S3 of the Supporting Information) exhibit pronounced peak-to-peak separations and distorted wave shapes. These features may be attributed to a combination of uncompensated resistance^46^ and heterogeneous distribution of conductive carbon black domains within the insulating PLA matrix that generates a partially blocked surface with nonuniform current distribution and heterogeneous diffusion domains.^63^ Such factors can lead to deviations from the ideal reversible voltammetric profile. Furthermore, conductive fillers partially embedded within the insulating PLA matrix may remain electrochemically inaccessible, contributing to the broader and less defined voltammetric profiles observed.^64^ Comparison between the preliminary CVs recorded at a single scan rate (Figures S2–S3) and CVs acquired over a range of scan rates (Figures S5–S10) indicates that successive potential cycling itself also induces electrochemical activation, as evidenced by the gradual approach of the *I*_*pa*_/*I*_*pc*_ ratio to unity.

Surface activation strongly affected the electrochemical behavior of the 3D-printed electrodes. In general, all activated electrodes exhibited markedly improved electrochemical performance compared to the untreated ones, confirming that surface modification effectively enhances electron transfer kinetics and overall electrode response, as exemplified by the CVs recorded at 50 mV s^–1^ in Figure 1A. The treated electrodes displayed higher *I*_*pa*_ and |*I*_*pc*_| and narrower Δ*E*_*p*_, while untreated electrodes exhibited the lowest peak currents and largest Δ*E*_*p*_, characteristic of sluggish heterogeneous electron transfer. This observation is further supported by EIS data (Figure 1B), which indicates a drastic reduction in the *R*_*ct*_ values for all treated samples. The untreated electrode exhibited the highest *R*_*ct*_ (18.03 ± 2.62 kΩ), whereas NaOH, NaOH-EC_NaOH_, and NaOH-EC_PBS_ resulted in the lowest *R*_*ct*_ values (0.28 ± 0.01 kΩ, 0.24 ± 0.05 kΩ, and 0.41 ± 0.06 kΩ, respectively). The corresponding *A*_*e*_ and *k* ^0^ are also summarized in Figure 1C,1D.

**Figure 1:**
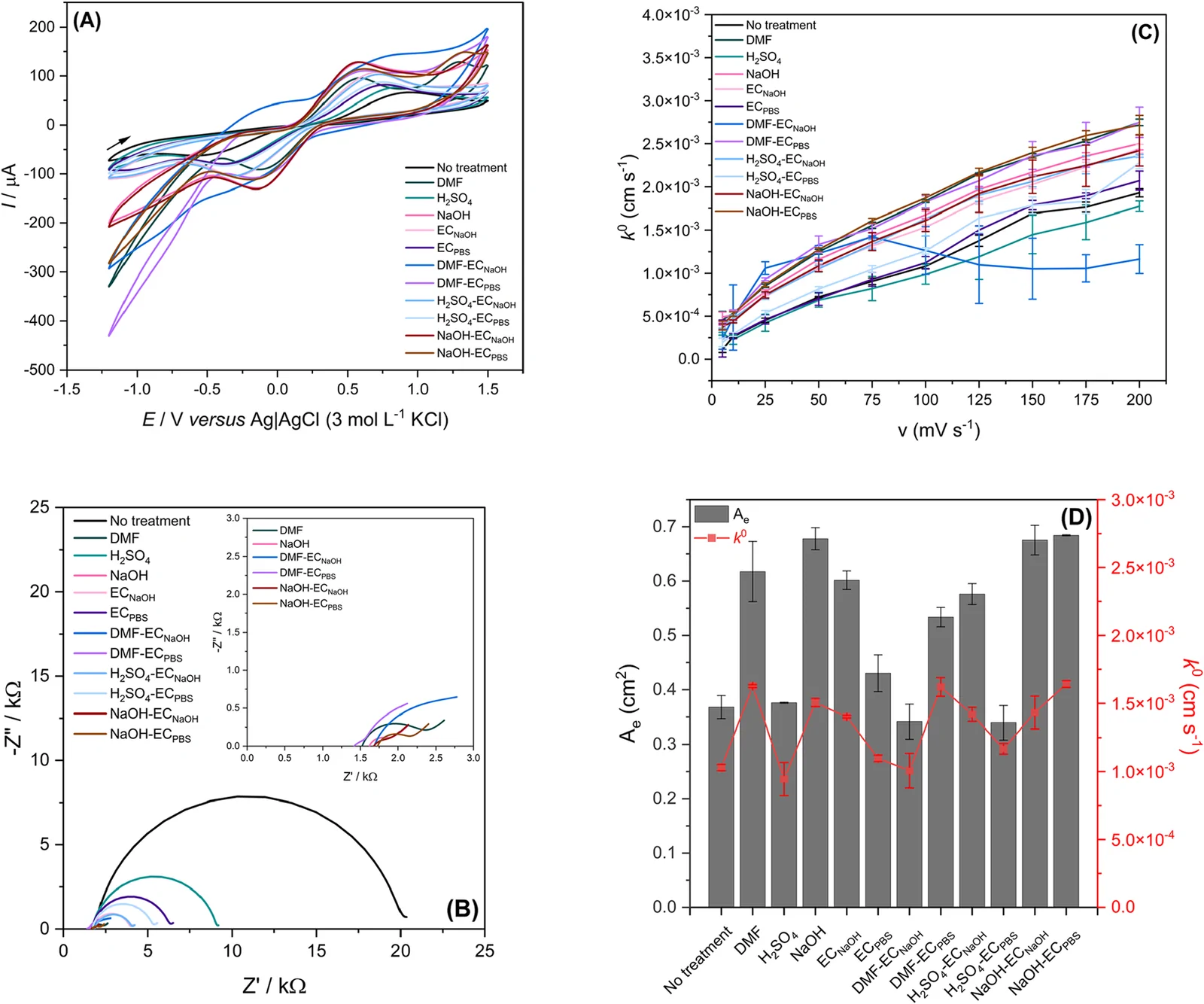
Electrochemical characterization of 3D-printed CB/PLA electrodes subjected to different activation treatments. (A) Cyclic voltammograms recorded at 50 mV s^–1^. (B) Nyquist plots obtained from electrochemical impedance spectroscopy (EIS) experiments. (C) Heterogeneous electron transfer rate constant (*k* ^0^) as a function of the scan rate (*v*), determined by the Klingler–Kochi method $\left(\overset{®}{{k^{0}}_{v}}\pm s\,\tan dard\;\,deviation\right)$ . (D) Mean electroactive area $(\overset{®}{A_{e}\;}\pm s\,\tan dard\;deviation,left\;y‐axis,\;grey\;\,bars)$ and heterogeneous electron transfer rate constant $\left(\overset{®}{k^{0}}\pm s\,\tan dard\;deviation,right\;y‐axis,red\,lines\;and\;squares\right)$ obtained from inter-replicate measurements for each treatment. For each electrode, *k* ^0^ represents the average of the ten scan-rate-dependent values (5–200 mV s^–1^). All measurements were performed in 1:1 mmol L^–1^ [Fe­(CN)_6_]^3–/4–^ in 0.1 mol L^–1^ KCl.

These improvements arise directly from the surface activation mechanisms, which modify the insulating PLA matrix and expose the conductive fillers. The chemical treatments employed operate via distinct mechanisms. The organic solvent DMF primarily acts by swelling and softening the PLA, disrupting the polymer cohesion and facilitating its partial dissolution.^5,35^ In contrast, the acidic (H_2_SO_4_) and basic (NaOH) solutions promoted hydrolysis of the ester bonds in the PLA polymer chain. In acidic media, the protonation of the carbonyl oxygen increases the electrophilicity of the carbonyl carbon, facilitating the nucleophilic attack by water. Under basic conditions, hydroxide ions directly act as nucleophiles, leading to base-catalyzed hydrolysis (saponification).^25^

Electrochemical activation is known to exfoliate the electrode surface and induce redox reactions that generate functional groups, thereby altering the surface polarity and enhancing electron transfer kinetics. Additionally, these treatments increase the density of defects and edge-plane sites, which contribute to an improved electrode performance.^35,65^ When combined with chemical treatments, electrochemical activation may complement chemical degradation mechanisms, also yielding electrodes with superior electrochemical performance compared to that of their as-printed counterparts.

The increase of Δ*E*_*p*_ with the scan rate observed across all samples (Figure S11 of the Supporting Information) likely reflects a combination of kinetic, morphological, and ohmic contributions. For 3D-printed electrodes, the heterogeneous distribution of conductive and insulating domains at the surface may be interpreted within the framework of partially blocked electrode theory, in which insulating regions act as blocking elements that generate heterogeneous diffusion domains, leading to nonuniform mass-transport conditions across the electrode surface.^66^ Such effects can alter the apparent reversibility of the electrochemical process and contribute to variability in voltammetric responses, even when the geometric surface area is unchanged. Although surface roughness and heterogeneity may increase the nominal electroactive area, the presence of partially blocked or poorly accessible regions can limit the effective electroactive area available for electron transfer.^66^

It is also important to note that uncompensated ohmic resistance (*R*_*u*_) contributes additively to Δ*E*_*p*_ independently of charge-transfer kinetics, as the potential difference due to ohmic drop equals the current passed times *R*_*u*_, and for large values of *R*_*u*_, the resulting ohmic drop affects the accuracy of the data.^46^ This is particularly relevant for 3D-printed electrodes, in which contact resistance arising from the electrical conductivity of the printed material represents an additional ohmic contribution to peak separation.^67^ Therefore, the Δ*E*_*p*_ values observed in the present study should be interpreted as the outcome of overlapping kinetic, morphological, and resistive phenomena.

The apparent increase of *k* ^0^ with the scan rate (Figure 1C) across most treatments can be explained by the mathematical structure of the Klingler–Kochi equation (eq ([Disp-formula eq2])), in which the √*v* term tends to dominate over the exponential dependence on Δ*E*_*p*_, even as Δ*E*_*p*_ itself increases with the scan rate due to the combined kinetic, morphological, and ohmic contributions discussed above. Consequently, the derived *k* ^0^ values should be interpreted as apparent kinetic constants. Their mean values (Figure 1D) were used solely for comparative evaluation of treatment efficiency rather than rigorous determinations of *k* ^0^, since Δ*E*_*p*_ no longer reflects exclusively charge-transfer kinetics under the present experimental conditions. Notably, DMF-EC_NaOH_ electrodes exhibited a maximum *k* ^0^ at intermediate scan rates (75 mV s^–1^), followed by a decline at higher rates. This behavior may be associated with the highly aggressive nature of the DMF-EC_NaOH_ treatment, which visibly removed a substantial amount of material from the electrode surface during the washing step, possibly increasing surface heterogeneity and amplifying non-kinetic contributions to Δ*E*_*p*_ at higher scan rates. Variations in α and Δ*E*_*p*_ with the scan rate also contribute to the observed dispersion of *k* ^0^ values.

Analysis of *A*_*e*_ revealed that NaOH, NaOH-EC_NaOH_, and NaOH-EC_PBS_ treatments yielded the highest mean values, possibly indicating the effective exposure of conductive regions. The electrodes that displayed the highest *A*_*e*_ values also generally exhibited superior *k* ^0^. Among the activated samples, H_2_SO_4_, DMF-EC_NaOH_, and EC_PBS_ treatments generated electrodes with the lowest *A*_*e*_ and *k* ^0^ values (Figure 1D), suggesting less efficient activation for H_2_SO_4_ and EC_PBS_ or, in the case of DMF-EC_NaOH_, possibly stronger deviations from the assumptions underlying eq ([Disp-formula eq1]), owing to the substantial surface modifications produced by this treatment. The nonlinear fitting results for the determination of *A*_*e*_ are summarized in Table S1 of the Supporting Information.

Although some treatments, such as H_2_SO_4_, DMF-EC_NaOH_, and H_2_SO_4_-EC_PBS_, led to higher peak currents, smaller Δ*E*_*p*_ values, and lower *R*_*ct*_ compared to the untreated samples, their *A*_*e*_ and *k* ^0^ were similar to the as-printed counterparts. This apparent discrepancy can arise from several factors. First, the enhancement in peak currents and charge-transfer kinetics may be primarily due to the improved exposure of conductive domains at the surface, rather than a substantial increase in the total electroactive area. In such cases, the reaction occurs more efficiently on existing active sites rather than on a significantly larger surface.

Second, the estimation of *A*_*e*_ from eq 1 assumes a reversible electrochemical process, mass transport exclusively by planar one-dimensional diffusion, and the absence of natural convection and migration contributions.^39,40,63^ These assumptions may not be fully satisfied for activated 3D-printed CB/PLA electrodes, which typically exhibit pronounced surface roughness, heterogeneous distributions of conductive domains, and complex microstructured morphologies. Recent work has tested the limits of the Randles–Ševčík equation using 3D-printed disk electrodes^63^ and demonstrated that deviations exceeding 100% can arise from radial diffusion and natural convection effects, particularly at slow scan rates, conditions relevant to the present study. Therefore, the *A*_*e*_ values reported should be interpreted primarily as comparative indicators of relative surface activation among treatments rather than as absolute measurements of the true electroactive area.

A similar limitation applies to the determination of *k* ^0^ using the Klingler–Kochi approach. The *k* ^0^ values obtained from the Klingler–Kochi method are sensitive to variations in Δ*E*_*p*_ and α,^44^ as already discussed, and may not capture local heterogeneities in electron transfer. Critically, since this method relies directly on Δ*E*_*p*_ to estimate *k* ^0^ and assumes that peak separation is governed predominantly by charge-transfer kinetics, any non-kinetic contribution to peak separation, including uncompensated contact resistance, ohmic drop within the composite material, surface heterogeneity, and nonuniform current distribution, will be incorporated into the derived rate constant.^67^ For this reason, even with visibly improved voltammetric and impedance responses, *A*_*e*_ and *k* ^0^ can remain within the same order of magnitude as those of untreated samples, and the *k* ^0^ values reported here, similarly to *A*_*e*_, should be used for relative comparison of treatment effectiveness rather than as rigorous determinations of the heterogeneous electron transfer rate constant.

Regarding the mass-transport mechanism, most electrodes subjected to the various treatments exhibited *R* ^2^ values close to unity in the *I*_*p*_ versus *v* ^1/2^ plots. However, electrodes treated with DMF-EC_NaOH_ (Figure S8 of the Supporting Information) showed the lowest coefficients (*R* ^2^ = 0.928 for *I*_*pa*_ and *R* ^2^ = 0.933 for *I*_*pc*_). This indicates that the electrochemical process, in this case, may not be purely diffusion-controlled but also influenced by the adsorption of electroactive species onto the electrode surface.^45,46^ Although these electrodes exhibited relatively high currents compared with some other treatments, significant peak broadening and loss of definition were observed across all scan rates. The visibly cloudy rinse water confirmed that the DMF-EC_NaOH_ protocol was highly aggressive, removing a considerable amount of material from the electrode surface, as discussed.

The Randles circuit was used to fit the EIS data, with χ^2^ values lower than 0.0001 in all cases, confirming the high quality of the fits. Nevertheless, electrodes treated with DMF-EC_PBS_ (Figure S8 of the Supporting Information) exhibited distinct behavior, with two semicircles in the Nyquist plot, indicating an additional resistance. In this case, the equivalent circuit shown in Figure S2.B was adopted. Both DMF-EC_NaOH_ and DMF-EC_PBS_ treatments led to the formation of cracks on the electrode surface. For the latter, the additional resistance is likely related to pores generated by treatment-induced deformations, which may have introduced an additional diffusion pathway and, consequently, a second semicircle in the Nyquist plots.^68^ For electrodes treated with DMF-EC_PBS_, a noticeable loss of peak definition was also observed in the CV profiles, particularly in *I*_*pa*_, resembling the behavior of electrodes treated with DMF-EC_NaOH_, which is likely associated with surface alterations induced by the treatment protocols. However, *R* ^2^ values close to unity in the *I*_*p*_ versus *v* ^1/2^ plots indicated that the electrochemical process remained predominantly diffusion-controlled.

### 3.2 Selection of Promising Treatments

To identify the most promising surface post-treatments, a combined metric *L* was utilized due to the necessity of a multicriteria comparison approach. Levene’s test (Table S2 of the Supporting Information), performed across different scan rates for each surface treatment, revealed that the variance of the individual electrochemical variables (*I*_*pa*_, |*I_pa_*/*I_pc_* | ratio, and Δ*E*_*p*_) exhibited some heterogeneity, with certain *p*-values below 0.05. These findings indicate that the assumptions required for conventional homoscedastic statistical procedures are not universally satisfied and, together with the observation that different electrochemical variables may rank activation protocols differently, highlight the limitations of relying on single-response analyses, which may vary in magnitude and reproducibility. Consequently, a composite score (*L*) was calculated by normalizing and equally weighting the variables, producing a single overall performance metric. This approach balances the influence of each variable, reduces dependence on any single-response variable, integrates complementary electrochemical information, and provides a more representative electrochemical performance of each electrode.

First, individual performance scores *L*_*t*,*v*,*r*_ were computed for each treatment *t*, scan rate *v*, and replicate *r* by combining normalized values of *I*_*pa*_, *I*_*pa*_/*I*_*pc*_, Δ*E*_*p*_, and *R*_*ct*_ (eq 7), using the Fe­(CN)_6_^3–/4–^ redox probe. In this approach, we studied the effect of *v* on *L*. The use of normalized variables ensures that the inherent differences in the units and scaling of these variables do not bias the overall performance ranking.

Inspection of the performance profiles across scan rates (Figure 2A) shows that, for most treatments, *L* is relatively stable as a function of scan rate: within-treatment fluctuations across the 10 investigated scan rates are small compared to differences between treatments. Levene’s test was conducted on *L* to assess the homogeneity of variances. When grouping by *v*, the variance of *L* was found to be homogeneous (*F* = 0.240, *p* = 0.99), suggesting that the kinetic modulation of the scan rate does not significantly introduce differential variability across the experimental range. However, when grouping *L* by surface treatment (data from all scan rates), the variance was found to be significantly heterogeneous (*F* = 5.038, *p* = 2.63 × 10^–7^). This result indicates that the chosen surface treatment is not only a determinant of the average performance (*L̅*) but also a major factor influencing the reproducibility and reliability of the 3D-printed electrodes.

**Figure 2:**
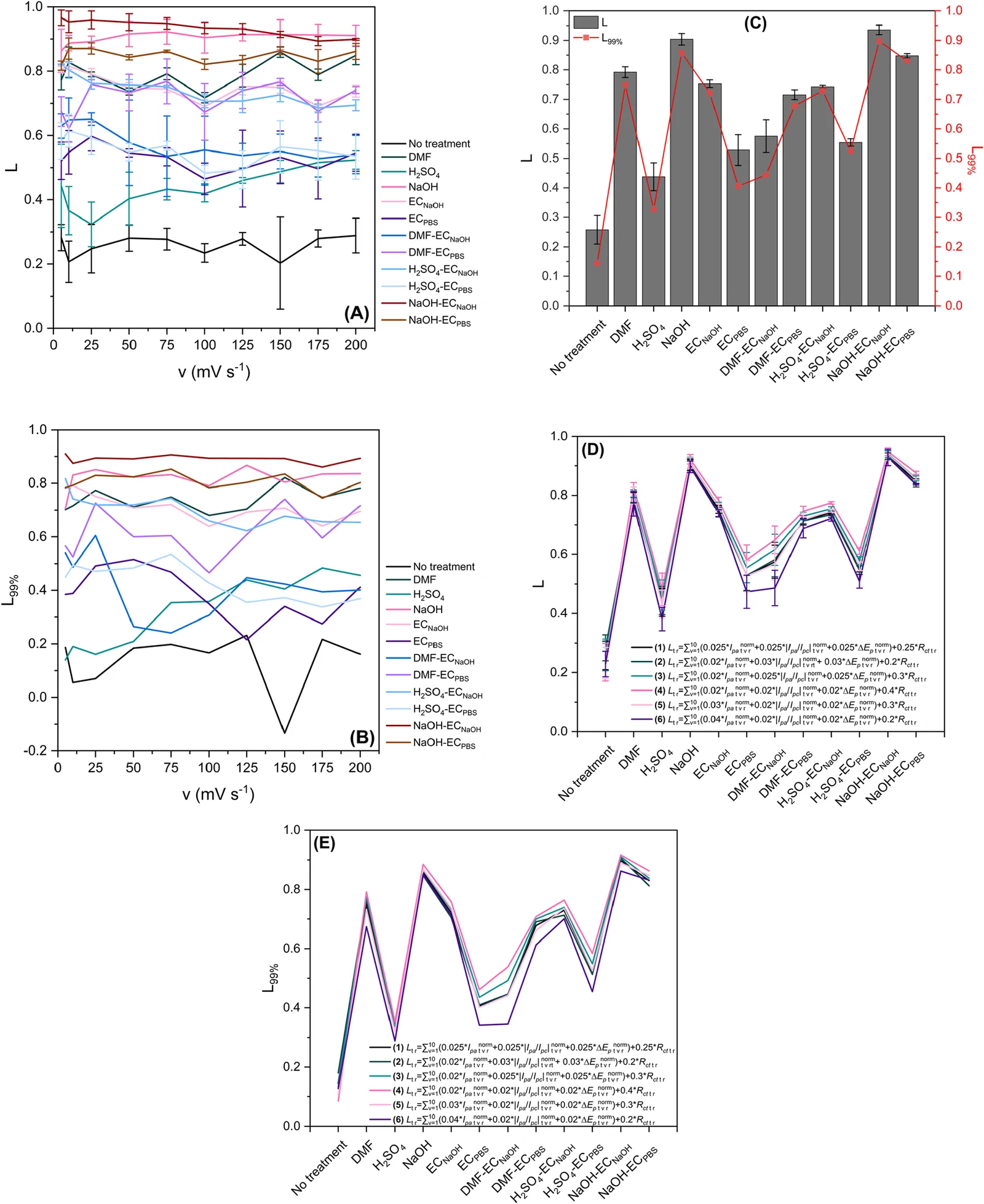
(A) Mean individual performance (*L̅* ± σ_*L*_) and (B) risk-adjusted performance (*L*_99%_) scores of 3D-printed electrodes as a function of scan rate for each surface treatment. (C) Global mean individual performance (*L̅* ± σ_*L*_) and risk-adjusted performance (*L*_99%_) scores for each surface treatment. Global comparison of the multiresponse indices (D) *L* and (E) *L*_99%_ functions for 3D-printed CB/PLA electrodes subjected to different surface treatments, considering all scan rates with differentiated variable weights.

Given the confirmed heterogeneity of variance among treatments and as a conservative measure, the risk-adjusted score (*L*_99%*t*,*v*_) was adopted as the primary comparison metric (eq ([Disp-formula eq8])). By explicitly penalizing treatments with high variability, *L*_99%_ (Figure 2B) provides a more robust and reliable ranking of electrode performance, effectively addressing the statistical limitations associated with unequal variances.

When the same analysis was extended to the risk-adjusted performance score, Levene’s test revealed that the variance of *L*_99%_ across scan rates remained homogeneous (*F* = 0.187, *p* = 0.995), confirming that the inclusion of the variability penalty did not introduce additional dispersion associated with the scan rate. However, when grouping by surface treatment, the variance of *L*_99%_ was significantly heterogeneous (*F* = 4.95, *p* = 3 × 10^–6^), indicating that treatment-dependent factors continue to dominate the reproducibility profile. This finding reinforces that the variability component incorporated into *L*_99%_ effectively captures reproducibility differences among treatments, distinguishing those that yield stable enhancements from those exhibiting less predictable performance.

Figure 2B displays *L*_99%_ for all treatments as a function of the scan rate. Among all of the conditions, the NaOH-EC_NaOH_ treatment consistently achieved the highest *L*_99%_ values across the entire scan rate range, indicating superior electrochemical activity combined with high reproducibility. From a practical standpoint, however, the single-step NaOH treatment also exhibited high and stable *L*_99%_ values, performing comparably to NaOH-EC_NaOH_, while requiring a simpler activation procedure. Thus, NaOH also represents a technically and operationally attractive alternative, balancing the performance, reproducibility, and process simplicity.

Although individual comparisons at each scan rate can be performed, such an approach does not capture the overall electrochemical behavior across the entire range. Therefore, a second methodology was adopted, in which the composite score (*L*_*t*,*r*_) integrated the contributions from all ten scan rates (eq 9). This cumulative value was then converted into a single risk-adjusted indicator (*L*_99%*t*_) (eq 10), allowing for a robust and statistically consistent comparison of overall electrode performance.

Figure 2C presents the results obtained using the integrated approach. The observed trends are fully consistent with the per-scan-rate analysis (Figure 2A,2B). Among all treatments, NaOH-EC_NaOH_ exhibited the highest *L* (0.94 ± 0.02) and *L*_99%_ (0.90) values, confirming its superior electrochemical performance and reproducibility. The NaOH treatment alone also displayed high *L* (0.90 ± 0.02) and *L*_99%_ (0.86) values, comparable to those of NaOH-EC_NaOH_, but with a simpler and more practical activation protocol. Following these two, NaOH-EC_PBS_ and DMF treatments achieved relatively high *L*_99%_ values (0.83 and 0.75, respectively), demonstrating their ability to enhance the electrochemical performance, even if their effect was less marked.

In contrast, the untreated electrodes showed the lowest *L* (0.26 ± 0.05) and *L*_99%_ (0.14) values, evidencing their poor electrochemical performance. This clearly demonstrates the crucial role of surface activation in improving the conductivity, exposure of active sites, and overall responsiveness of 3D-printed electrodes. The remaining treatments showed intermediate performance, indicating partial improvement but insufficient activation compared to the alkaline protocols.

An important observation emerging from the *L*_99%_ analysis is that electrochemical activation in either NaOH or PBS was not among the highest-performing strategies, despite its widespread use in the literature.^33^ The continued popularity of electrochemical activation protocols likely reflects not only their effectiveness but also practical advantages such as operational simplicity, low cost, accessibility, and implementation using conventional electrochemical instrumentation and dilute electrolytes, unlike chemical treatments that often require concentrated reagents or organic solvents. EC_NaOH_ and EC_PBS_ yielded *L*_99%_ values of 0.72 and 0.41, respectively, whereas the NaOH treatment alone achieved a higher score (0.86). These results suggest that the performance gains commonly associated with alkaline electrochemical activation may arise not only from the electrochemical process itself but also from chemical modifications induced by the alkaline medium, particularly, the partial hydrolysis of the PLA matrix. The lower performance of EC_PBS_ treatment further supports this interpretation, as the absence of an alkaline medium restricts the activation mechanism to electrochemically induced surface modifications without the contribution of PLA hydrolysis. Accordingly, alkaline electrochemical treatments may be viewed as the combined action of electrochemical activation and PLA saponification under polarized conditions. Future studies employing complementary physicochemical characterization techniques will be essential to elucidating the relative contributions of electrochemical treatment and alkaline-induced polymer modification to the overall activation process.

In this study, all variables were equally weighted in this multiresponse approach, assuming equal importance for each variable in the overall electrode performance. To investigate the impact of differential prioritization, the weights assigned to each variable in the integrated approach were varied between 0.2 and 0.4 during the comparison step. These variations represent different analytical emphasis scenarios, such as prioritizing improved charge transfer or enhanced reversibility, depending on application-specific demands.

As illustrated in Figure 2D,2E, the comparison between the models revealed that the overall profiles of *L* and *L*_99%_ remained highly consistent across all treatments when changing the weights. Small vertical shifts were observed, but the ranking among treatments was unchanged. Treatments that ranked highest under equal weighting (NaOH-EC_NaOH_, NaOH, and NaOH-EC_PBS_) maintained their superior positions regardless of how weights were varied, and the relative order among intermediate and lower-ranked treatments was preserved as well.

This consistency confirms that the multiresponse composite metric robustly reflects the overall performance of the electrodes. Hence, the results reinforce the credibility of this weighted multiresponse framework for objectively selecting optimal surface treatment protocols, providing flexibility to adapt weighting schemes to match targeted sensor performance requirements without affecting the choice of the best protocol. In practical terms, applying differentiated weights refines the quantitative assessment of electrode performance without altering the overall interpretation, thereby reinforcing the reliability of *L*_99%_ as an optimization criterion for electrochemical (bio)­sensing.

In general, a positive relationship was found between the multiresponse index *L*_99%_ (Figure 2C) and both *A*_*e*_ and *k* ^0^ (Figure 1D). Treatments exhibiting higher *L*_99%_ values also tended to present larger *A*_*e*_ values and faster electron-transfer kinetics, whereas those with lower *L*_99%_ values showed reduced surface area and slower charge transfer. Minor deviations from this trend may arise from local variations in surface morphology, heterogeneous exposure of conductive domains, or experimental factors affecting the estimation of *A*_*e*_ and *k* ^0^. Overall, this relationship reinforces the comprehensiveness of the *L*_99%_ index in reflecting the key aspects of electrode activation and performance.

### 3.3 Electrical Resistance

The electrical resistance measurements used in this study provide an indirect approach to assessing the impact of each surface treatment on electrode conductivity. Since electrical resistance is directly related to resistivity, which, in turn, is inversely proportional to conductivity, a reduction in resistance for electrodes with similar lengths and cross-sectional areas indicates an increase in conductivity.^52^

The potential–current plots (Figure 3A) were analyzed by linear regression, following Ohm’s law, to determine the electrical resistance. All regressions exhibited excellent linearity (*R* > 0.9995), confirming the ohmic behavior of the 3D-printed electrodes. The detailed regression parameters, including slope estimates, standard errors, and correlation coefficients for each replicate, are summarized in Table S3 of the Supporting Information, while the mean resistance values are plotted in Figure 3B.

**Figure 3:**
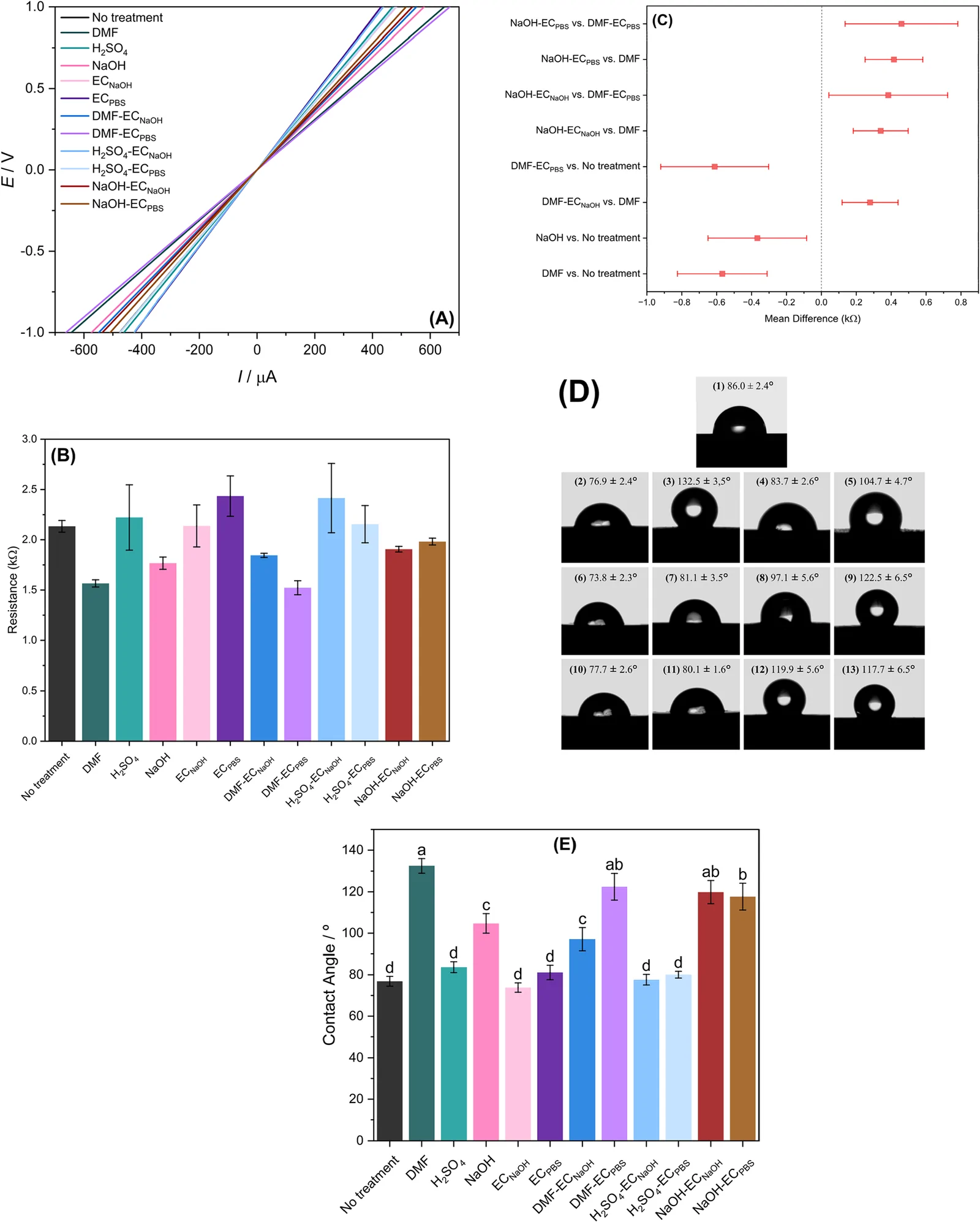
(A) Representative potential (*E*) versus current (*I*) plots for 3D-printed electrodes subjected to different surface treatments (50 mV s^–1^), illustrating the linear relationship of Ohm’s law (*E* = *RI*). (B) Mean electrical resistance of 3D-printed electrodes subjected to different surface treatments, showing inter-replicate standard deviation. (C) Mean differences (±95% confidence interval) in electrical resistance among significant treatments only (Games-Howell, Welch’s ANOVA; α = 0.05). (D) Contact angles: (1) CB/PLA, (2) No treatment, (3) DMF, (4) H_2_SO_4_, (5) NaOH, (6) EC_NaOH_, (7) EC_PBS_, (8) DMF-EC_NaOH_, (9) DMF-EC_PBS_, (10) H_2_SO_4_-EC_NaOH_, (11) H_2_SO_4_-EC_PBS_, (12) NaOH-EC_NaOH_, and (13) NaOH-EC_PBS_. (E) Tukey’s test (α = 0.05) for contact angles of the 3D-printed electrodes (mean ± standard deviation).

Before comparing the treatments, Levene’s test for homogeneity of variances was performed (*F* = 6.37; *p* = 7.8 × 10^–5^), revealing that the variances among groups were significantly different (*p* < 0.05). Consequently, Welch’s ANOVA, which does not assume homoscedasticity, was applied. This test confirmed the existence of significant differences among the mean resistance values of the treatments (*F* = 23.40; *p* = 2.8 × 10^–5^). To identify which treatments differed statistically, pairwise Games-Howell comparisons were conducted at a significance level of 0.05 (Figure 3C).

The results confirm that surface activation markedly affects the conductivity of the CB/PLA electrodes. DMF and DMF-EC_PBS_ treatments yielded the lowest average resistances (1.57 ± 0.04 and 1.52 ± 0.07 kΩ, respectively), suggesting removal of insulating PLA domains. The NaOH, NaOH-EC_NaOH_, and NaOH-EC_PBS_ treatments also led to substantially lower resistances (1.77 ± 0.06, 1.91 ± 0.03, and 1.98 ± 0.03 kΩ, respectively) compared with the untreated electrodes (2.13 ± 0.06 kΩ), confirming the effectiveness of alkaline activation.

The pairwise comparisons summarized in Figure 3C provide further insight into these trends. The results reveal that the listed pairs exhibit nonoverlapping confidence intervals with zero, confirming significant differences among them. NaOH, DMF, and DMF-EC_PBS_ treatments exhibited significant negative mean differences relative to the untreated electrodes (*p* < 0.05), confirming their effectiveness in reducing electrical resistance. DMF-EC_NaOH_, in turn, exhibited a positive mean difference versus that of DMF (mean difference = 0.279; *p* = 0.0096), indicating that its resistance was slightly but significantly higher than that of DMF. Similarly, the combined alkaline-electrochemical treatments (NaOH-EC_NaOH_ and NaOH-EC_PBS_) displayed positive mean differences when compared with DMF-based activations, confirming that they possess slightly higher Ohmic resistance despite their high overall activation efficiency.

When related to the *L*_99%_ index, it becomes evident that the treatments achieving the lowest resistances (DMF and DMF-EC_PBS_) do not necessarily deliver the highest *L*_99%_ values (0.68 and 0.75, respectively), revealing that conductivity alone does not ensure an optimal electrochemical performance. Conversely, the alkaline-based activations (NaOH, NaOH-EC_NaOH_, and NaOH-EC_PBS_) combined moderately low resistances with the highest *L*_99%_ values. This suggests that alkaline activation not only enhances conductivity but also promotes chemical functionalization and exposure of electroactive sites, which are essential for efficient charge transfer. Furthermore, these treatments exhibited low standard deviations in resistance, indicating uniform and reproducible conductive pathways, which are critical for a consistent electrochemical (bio)­sensing performance.

Although differences in aggressiveness among treatments could induce slight changes in the electrode cross-sectional area, potentially influencing resistance according to Ohm’s second law, no pronounced macroscopic changes in electrode thickness were observed. Hence, any geometric variations were considered negligible, and the electrical resistance measurements were deemed valid and comparable among treatments.

### 3.4 Contact Angles

Figure 3D shows one of the images obtained from the triplicates used to determine the contact angle, along with the mean and inter-replicate standard deviation of the measured angles. Levene’s test indicated that the variances of the contact angle measurements were homogeneous (*F* = 1.29, *p* = 0.29 > 0.05), satisfying one of the assumptions for parametric analysis. The Shapiro–Wilk test, despite the low statistical power for very small samples (*n* = 3 per treatment), indicated no significant deviation from normality for any treatment (*W* = 0.773–0.999, *p* = 0.051–0.93). Thus, the assumptions for parametric testing were considered fulfilled. Considering sample independence and approximate normality, a one-way ANOVA (*p* < 0.05) followed by Tukey’s post hoc test was applied to compare the surface wettability among treatments (Figure 3E).

Filament data were excluded from statistical analysis, as the focus was on variation due to electrode treatments. The filament exhibited a contact angle of 86.0° ± 2.3°, whereas untreated electrodes had a contact angle of 76.9° ± 2.4°. This slight reduction in the contact angle of the untreated electrode compared to the filament likely reflects greater PLA deposition on the electrode surface during extrusion, considering that PLA is more hydrophilic than CB.^69^

Figure 3E demonstrates that the treatments DMF, NaOH-EC_NaOH_, DMF-EC_PBS_, NaOH-EC_PBS_, NaOH, and DMF-EC_NaOH_ (groups “a”, “b”, and “c”) significantly increased contact angles in relation to the untreated electrodes. The enhanced hydrophobicity produced by these treatments reflects more effective removal of PLA and greater exposure of the conductive filler. Specifically, DMF acts as a solvent for PLA,^35,70^ while H_2_SO_4_ and NaOH facilitate polymer hydrolysis.^25^ Electrochemical procedures promote surface exfoliation and the formation of new functional groups,^35,71^ as previously discussed.

The highest *L*_99%_ values (NaOH-EC_NaOH_, NaOH, NaOH-E_CPBS_, and DMF) align with treatments that also present higher contact angles. However, treatments labeled with the letter “d” showed improved electrochemical performance (*L*_99%_) relative to the untreated electrodes (Figure 2C) but no statistically significant change in contact angle compared to the as-printed samples. This suggests that optimal electrochemical performance and reproducibility arise not solely from maximizing hydrophobicity, but from a balanced combination of surface wettability modification and effective chemical/electrochemical activation.

It is important to note that contact angle measurements are inherently influenced by the surface morphology. As 3D-printed electrodes have naturally rough surfaces, and treatments may introduce additional topographical features or inhomogeneities, observed contact angles reflect not only changes in surface chemistry but also alterations in surface roughness and physical structure induced by the various post-treatments.

### 3.5 Scanning Electron Microscopy with Energy-Dispersive X-ray Spectroscopy (SEM-EDS) Analyses

To evaluate the effects of the treatments on the electrode surface and understand their impact, SEM-EDS analyses were performed. The obtained images are shown in Figures S12–S15 of the Supporting Information. Representative SEM micrographs of selected samples acquired at 100× and 40,000× magnifications are also shown in Figure 4A, while the EDS spectra and elemental maps for both the filament cross section and the NaOH-treated samples are presented in Figure 4B,4C.

**Figure 4:**
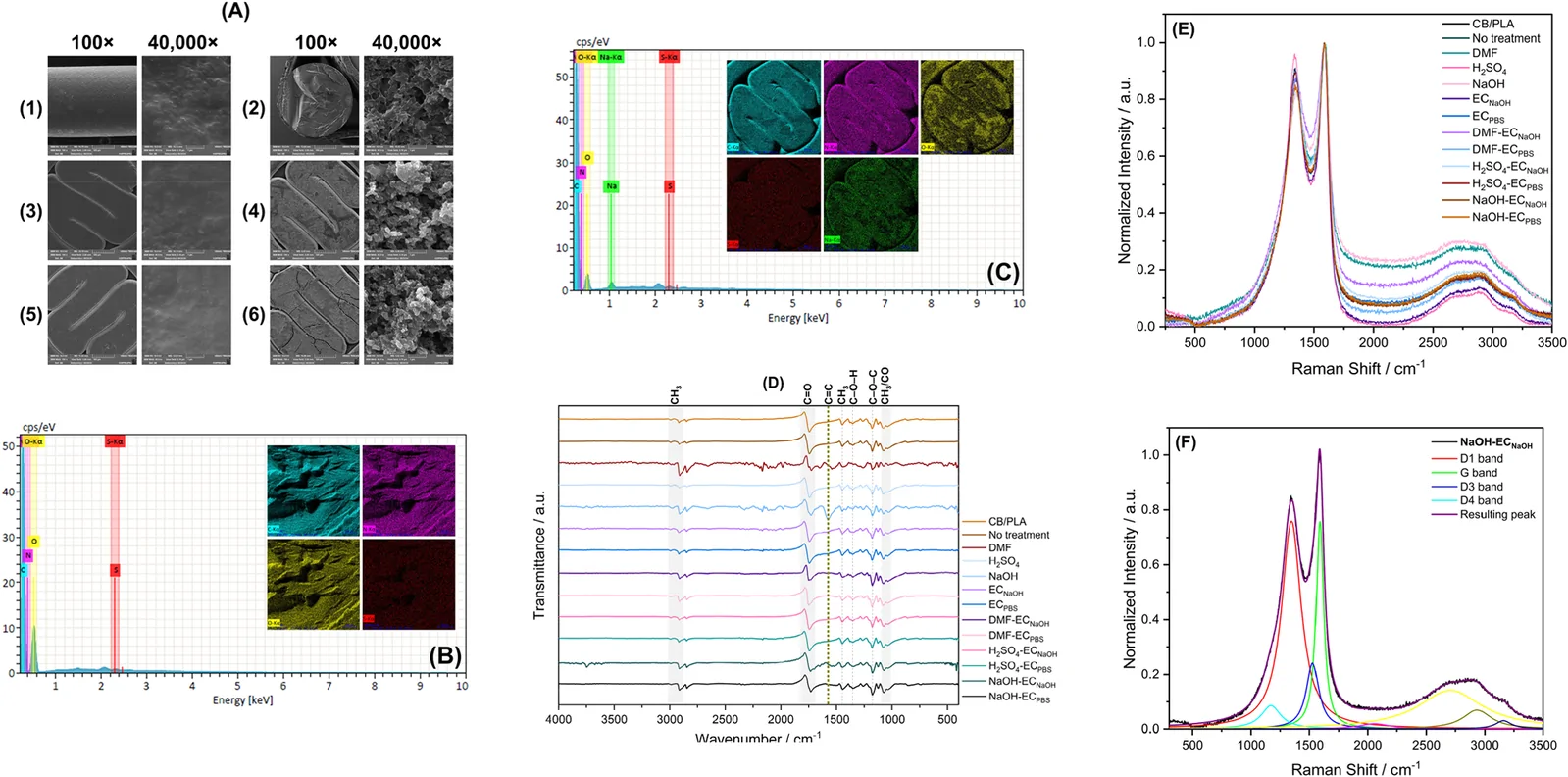
(A) SEM images of selected samples acquired at 100× (left) and 40,000× (right): (1) CB/PLA filament, (2) CB/PLA cross section, (3) untreated electrode, (4) NaOH-treated electrode, (5) EC_PBS_-treated electrode, and (6) DMF-EC_NaOH_-treated electrode. EDS spectrum and elemental distribution maps for the filament cross section (B) and the NaOH-treated electrode (C). (D) FTIR spectra of the filament, untreated electrode, and electrodes subjected to the different activation methods. (E) Raman spectra of the CB/PLA filament and the 3D-printed electrodes before and after the different activation treatments. (F) Example of the deconvoluted Raman spectrum for the NaOH-EC_NaOH_-treated electrode (*R* ^*2*^ = 0.998), showing the fitted components.

Analysis of Figure 4A.1 and 4A.3 reveals that both the CB/PLA filament and the untreated electrode exhibit relatively smooth and uniform surfaces with no major deformations. Chemical treatments, on the other hand, introduced surface irregularities and varying levels of roughness (Figure 4A.4). Among these, H_2_SO_4_ treatment produced the least deformation. These results are also confirmed by Figures S12 and S13 of the Supporting Information.

Electrochemical treatments (Figure 4A.5) resulted in less pronounced roughness, promoting light surface exfoliation. Combined treatments, as shown in Figure 4A.6, introduced several surface defects. However, electrodes treated with H_2_SO_4_ again displayed a smoother surface. This information is highlighted by Figures S14 and S15 of the Supporting Information.

The fact that not all treatments producing extensive surface deformities resulted in the highest *L*_99%_ values (Figure 2C) highlights the complex interplay between surface morphology and electrochemical performance. While the best treatments (NaOH-EC_NaOH_, NaOH, NaOH-E_CPBS_, and DMF) displayed multiple surface deformities and enhanced electrochemical performance, others with fewer morphological changes (H_2_SO_4_-EC_NaOH_ with *L*_99%_ = 0.73, and EC_NaOH_ with *L*_99%_ = 0.72) also showed good, but not top, performance. Conversely, some treatments with minimal surface irregularities (H_2_SO_4_ with *L*_99%_ = 0.33 and EC_PBS_ with *L*_99%_ = 0.41) exhibited poorer electrochemical behavior. Nevertheless, excessive etching did not always lead to an improved performance. The DMF-EC_NaOH_ treatment, for instance, generated severe cracking on the electrode surface (Figures 4A.6 and S15) and yielded only a moderate *L*_99%_ value (0.44). In contrast, the DMF-EC_PBS_ treatment (*L*_99%_ = 0.68) (Figure S15) caused milder cracking and maintained better electrochemical performance than DMF-EC_NaOH_. This suggests that beyond a certain threshold, aggressive activation can compromise the mechanical integrity and electron-transfer pathways.

EDS analysis of the CB/PLA filament cross section (Figure 4B) revealed the presence of carbon (47.29%), nitrogen (20.67%), oxygen (31.98%), and trace sulfur (0.06%). These elements were therefore monitored across all analyzed electrodes along with elements introduced by the treatment solutions. By partially removing the insulating PLA layer and exposing the conductive CB domains, the treatments were expected to increase the carbon atomic percentage (C %) while decreasing that of oxygen (O %), since CB is mainly composed of carbon, whereas PLA has a higher oxygen content due to its ester functionalities (Figure S16 of the Supporting Information). Table 2 presents the mass percentages of carbon and oxygen obtained by EDS for the CB/PLA filament and for the electrodes after each treatment.

**Table 2:** Mass Percentage of Carbon and Oxygen Obtained by EDS for the CB/PLA Filament and 3D-Printed Electrodes Subjected to Different Treatments

sample	C (%)	O (%)
CB/PLA	47.29	31.98
No treatment	47.39	31.75
DMF	55.98	12.54
H_2_SO_4_	46.97	32.09
NaOH	54.01	16.51
EC_NaOH_	47.70	31.34
EC_PBS_	47.56	31.41
DMF-EC_NaOH_	55.08	14.49
DMF-EC_PBS_	54.74	15.54
H_2_SO_4_-EC_NaOH_	46.89	32.24
H_2_SO_4_-EC_PBS_	56.16	34.79
NaOH-EC_NaOH_	53.65	19.61
NaOH-EC_PBS_	46.75	32.52

The reference samples (CB/PLA filament and untreated electrode) exhibited similar percentages of carbon and oxygen, reflecting the original material composition, where a substantial amount of PLA is still present. Among the individual chemical treatments, DMF and NaOH proved to be most effective at increasing the carbon content while reducing oxygen levels, suggesting successful exposure of conductive domains. Conversely, H_2_SO_4_ produced minimal change in the C/O ratio, consistent with its limited etching capability observed in SEM (see Figure S13 of the Supporting Information).

Isolated electrochemical treatments (EC_NaOH_ and EC_PBS_) did not noticeably alter the C/O ratio compared to the untreated sample, indicating that electrochemical activation alone is insufficient to remove the polymer matrix effectively. In contrast, combined chemical-electrochemical treatments such as DMF-EC_NaOH_, DMF-EC_PBS_, and NaOH-EC_NaOH_ substantially increased the carbon fraction and decreased oxygen, suggesting efficient removal of surface PLA and exposure of CB. Interestingly, the NaOH-EC_PBS_ treatment, despite visible surface modification in SEM (Figure S15 of the Supporting Information), did not markedly change the C/O ratio, possibly due to the formation of oxygenated functional groups. Similarly, H_2_SO_4_-EC_NaOH_ maintained a C/O ratio close to that of the untreated sample, while H_2_SO_4_-EC_PBS_ exhibited simultaneous increases in both carbon and oxygen, suggesting oxidation and incorporation of oxygenated species.

In general, the EDS findings were consistent with previous discussions. Treatments showing the best electrochemical performances, particularly NaOH and NaOH-EC_NaOH_, also exhibited an effective exposure of CB, confirming their suitability as activation protocols for 3D-printed CB/PLA electrodes. However, the DMF-EC_NaOH_ treatment, despite promoting extensive surface etching and higher carbon exposure, was excessively aggressive and likely compromised the structural integrity, resulting in only moderate electrochemical performance.

### 3.6 Attenuated Total Reflection Fourier Transform Infrared (ATR-FTIR)

The FTIR spectra of the CB/PLA filament, the untreated electrode, and the activated samples are shown in Figure 4D. Characteristic vibrations associated with PLA were observed, including asymmetric (2910–2916 cm^–1^) and symmetric (2987–2991 cm^–1^) CH_3_ stretching, as well as bands from oxygenated groups, such as C = O stretching (1730–1743 cm^–1^), C–O–H (1351–1353 cm^–1^), C–O–C stretching (1174–1178 cm^–1^), and CH_3_ rocking/C–O–C overlapping bands (1031–1078 cm^–1^). Additional bands at 1444–1446 cm^–1^ and 847–860 cm^–1^ were attributed to CH_3_ bending and C–C stretching, respectively.^29,72,73^

Oxygenated and nonoxygenated functional groups from PLA may interact with CB structures, resulting in overlaps, shifts, or broadening of CB-related vibrational bands. These interactions obscure the distinction between CB and PLA features in the FTIR spectra.^72^ In the spectrum of the electrode chemically treated with NaOH, a medium-intensity band appeared between 1566 and 1650 cm^–1^ (centered at 1576 cm^–1^), associated with C = C stretching of cyclic alkenes,^74^ likely arising from the CB structure (Figure S16 of the Supporting Information). This observation supports the hypothesis that alkaline treatment promotes greater CB exposure on the electrode surface, consistent with electrochemical results identifying NaOH as one of the most effective activation methods.

### 3.7 Raman Spectroscopy

Raman spectroscopy was employed to investigate the effects of surface post-treatments on the structure of the CB/PLA electrodes. All acquired spectra exhibit the characteristic *D* band (∼1347 cm^–1^) and *G* band (∼1590 cm^–1^). The *D* band is associated with structural defects such as vacancies, grain edges, heteroatoms, and *sp* ^*3*^-type hybridization, while the *G* band corresponds to the E_2g_ mode of *sp* ^2^ carbon, representing the in-plane vibration of carbon–carbon bonds in graphitic layers.^35,75,76^ A second-order vibrational mode, the *2D* band (∼2700 cm^–1^), related to electronic dispersion in graphitic carbon, is also observed,^21,76^ as shown in Figure 4E.

For detailed analysis, the Raman spectra were baseline-corrected, normalized, and deconvoluted using Lorentzian functions. The *D* and *G* bands were fitted with two to five peaks,^59^ while the *2D* band was fitted with three.^77^ The best fit (highest *R* ^2^) was obtained with four components for the *D* and *G* bands, designated as *D1* (∼1346 cm^–1^), *G* (∼1589 cm^–1^), *D3* (∼1520 cm^–1^), and *D4* (∼1180 cm^–1^), consistent with previous reports for carbon black.^78,79^ An additional fitted component between the *G* and *2D* bands was included in all spectra to improve the overall fit quality without altering the main peak positions (magenta curve in Figure 4F). All fits achieved *R* ^2^ values greater than 0.997, except for the H_2_SO_4_-treated electrode (*R* ^2^ = 0.989). An exemplary deconvolution for the NaOH-EC_NaOH_-treated electrode is presented in Figure 4F.

The *D1* band corresponds to disordered graphitic domains, while the *G* band represents the more crystalline *sp* ^*2*^-carbon structure. The *D3* band arises from the amorphous carbon fraction, and the *D4* band is attributed to highly disordered graphitic layers associated with ionic impurities and polyenic chains.^79^ Although there are similar vibrational features across all samples, noticeable variations in band intensity and width were observed (Figure 4E). The intensity ratio *I*_*D*_/*I*_*G*_, a key indicator of defect density in carbon materials, and the full width at half-maximum (FWHM) of the *D* and *G* bands revealed differences among treatments (Table 3). Both *I*_*D*_/*I*_*G*_ and FWHM of these bands are closely linked to the carbon microstructure. In general, an increase in *I*_*D*_/*I*_*G*_, as well as in FWHM of the *D* and *G* bands, typically indicates a higher concentration of defects and structural disorder.^59,80^

**Table 3:** Deconvolution Results of Raman Spectra for the Analyzed Samples^a^

sample	*D1* band position (cm^–1^)	*G* band position (cm^–1^)	*I*_*D*_/*I_G_*	*D1* band FWHM (cm^–1^)	*G* band FWHM (cm^–1^)
CB/PLA	1348.1	1596.2	0.979	166.83	70.67
No treatment	1341.8	1586.4	1.010	182.44	78.32
DMF	1349.7	1592.5	0.923	172.47	81.33
H_2_SO_4_	1340.1	1585.5	1.028	164.70	69.05
NaOH	1352.8	1592.4	1.089	203.13	84.32
EC_NaOH_	1343.4	1586.8	0.977	180.10	72.03
EC_PBS_	1345.5	1588.7	0.994	190.66	77.72
DMF-EC_NaOH_	1354.3	1590.2	1.127	219.18	86.02
DMF-EC_PBS_	1344.8	1588.1	1.044	193.92	77.98
H_2_SO_4_-EC_NaOH_	1350.6	1591.8	1.043	222.54	76.32
H_2_SO_4_-EC_PBS_	1341.4	1586.2	1.004	184.32	78.21
NaOH-EC_NaOH_	1346.9	1589.2	1.002	204.95	77.05
NaOH-EC_PBS_	1346.3	1589.7	0.985	203.27	78.50

a FWHM: full width at half-maximum.

The FWHM of the *D1* band increased after some treatments, indicating increased structural defects. The untreated electrode showed a *D1* FWHM of 182.44 cm^–1^, while the highest values were observed for the H_2_SO_4_-EC_NaOH_ and DMF-EC_NaOH_ treatments (222.54 cm^–1^ and 219.18 cm^–1^, respectively), indicating a more disordered structure. However, these treatments did not yield correspondingly high electrochemical performance, as evidenced by their moderate *L*_99%_ of 0.73 and 0.44, respectively. The most promising treatments (NaOH, NaOH-EC_NaOH_, and NaOH-EC_PBS_) exhibited intermediate *D1* FWHM values of 203.13 cm^–1^, 204.95 cm^–1^, and 203.27 cm^–1^, respectively.

Broadening of the *G* band FWHM was also observed in certain treated electrodes, suggesting increased disorder, especially for DMF-EC_NaOH_ (86.02 cm^–1^). NaOH, NaOH-EC_NaOH_, and NaOH-EC_PBS_ showed intermediate values of 84.32 cm^–1^, 77.05 cm^–1^, and 78.50 cm^–1^, respectively, close to the value observed for the untreated electrode (78.32 cm^–1^).

Similarly, the *I*_*D*_/*I*_*G*_ ratio did not exhibit a direct correlation with the electrochemical performance but still provided useful insights. The untreated electrode presented a ratio of 1.010, indicating a moderate degree of disorder. DMF-EC_NaOH_ exhibited the highest ratio (1.127), indicating an increased disorder. In contrast, the NaOH, NaOH-EC_NaOH_, and NaOH-EC_PBS_ treatments yielded ratios of 1.089, 1.002, and 0.985, respectively; the NaOH treatment exceeded the untreated value, whereas the other two were slightly lower. DMF treatment alone presented a reduced ratio (0.923), suggesting mild structural reordering, consistent with observations by Kalinke et al.^35^ for PLA-graphene electrodes chemically treated with DMF for 10 min.

In summary, chemical and electrochemical treatments induce structural modifications in the carbon black phase of 3D-printed electrodes. However, an increase in structural disorder does not necessarily correlate with an enhanced electrochemical performance. While Raman spectroscopy reveals the presence and degree of defects in the carbon structure, this technique alone cannot determine whether these defects improve the electrical conductivity or generate additional electroactive sites. For instance, exposure of edge-plane or defect sites can enhance kinetics, whereas overetching or excessive disorder may disrupt conductive pathways, ultimately limiting electrochemical performance.^81,82^

### 3.8 Thermogravimetric Analysis (TGA)

TGA was performed on the filament and the circular sensing region of the working electrode, both treated and untreated, as these regions correspond to the area used in electrochemical measurements. The analysis aimed to characterize thermal decomposition profiles, confirm CB incorporation in the filament, and assess the impact of surface post-treatments on electrode thermal stability (Figure 5A). The onset degradation temperature (*T*_*onset*_) and peak degradation temperature (*T*_*peak*_) were recorded (Table 4), with *T*_*peak*_ defined as the minimum point of the first derivative of the mass-loss curve. A pure PLA filament was analyzed as a reference.

**Figure 5:**
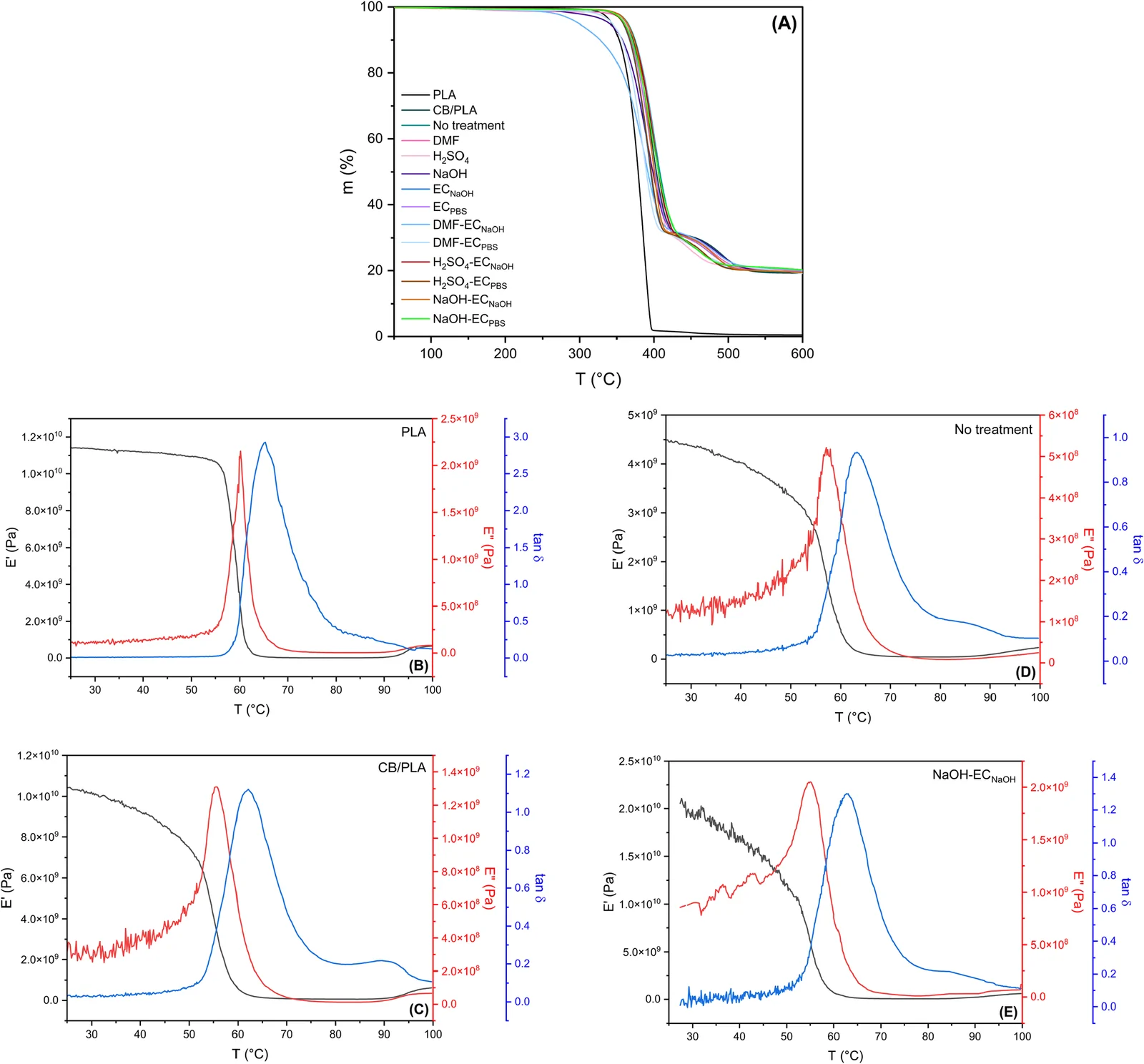
(A) Thermogravimetric decomposition curves of PLA, CB/PLA filament, untreated electrode, and electrodes subjected to different surface post-treatments. Dynamic mechanical analysis (*E*′, *E″*, and *tan δ*) curves for the following samples: (B) pure PLA filament, (C) CB/PLA filament, (D) untreated electrode, and (E) NaOH-EC_NaOH_-treated electrode.

**Table 4:** Data Obtained from TGA Experiments

sample	*T*_*onset*_ (°C)	*T*_*peak1*_ (°C)	*T*_*peak2*_ (°C)	*%m*_*final*_
PLA filament	349.74	385.85		0.48
CB/PLA filament	366.88	396.99	491.67	19.22
No treatment	370.88	399.81	491.35	19.53
DMF	369.71	402.67	478.62	19.99
H_2_SO_4_	364.22	396.98	452.88	20.19
NaOH	351.91	390.28	490.48	19.74
EC_NaOH_	356.43	391.49	470.17	19.60
EQPBS	360.54	393.30	476.53	19.66
DMF-EC_NaOH_	340.03	388.89	479.91	20.04
DMF-EC_PBS_	347.76	387.06	470.72	19.14
H_2_SO_4_-EC_NaOH_	370.26	399.49	452.39	20.25
H_2_SO_4_-EC_PBS_	360.04	392.39	459.18	19.41
NaOH-EC_NaOH_	369.60	398.85	483.99	19.52
NaOH-EC_PBS_	357.49	397.28	457.49	20.23

The pure PLA filament exhibited *T*_*onset*_ of 349.74 °C and a single peak degradation temperature (*T*_*peak1*_) of 385.85 °C, corresponding to the temperature at which degradation is maximal, with nearly complete decomposition and minimal residue (<0.50%). In contrast, the commercial CB/PLA filament began to decompose at 366.88 °C, indicating higher thermal stability, probably due to the presence of CB. CB/PLA displayed two degradation peaks (*T*_*peak1*_ = 396.99 °C and *T*_*peak2*_ = 491.67 °C), consistent with the manufacturer’s safety data sheet, which reports the presence of other polymers, probably used as plasticizers, in its composition. The residual mass of 19.22% at 600 °C aligns with the manufacturer’s reported CB content of less than 20% and is in agreement with literature values.^83^

Fabrication of the electrodes by 3D printing slightly increased both *T*_*onset*_ and *T*_*peak1*_, while the second peak temperature (*T*_*peak2*_) remained nearly unchanged, indicating subtle modifications of the PLA thermal behavior during processing. Among the chemical treatments, NaOH produced the most pronounced reduction in both *T*_*onset*_ and *T*_*peak1*_, followed by H_2_SO_4_ and DMF, indicating that alkaline hydrolysis has the strongest effect on the PLA degradation. The second peak, primarily related to the degradation of polymer additives, was more affected by H_2_SO_4_, followed by DMF and NaOH.

Electrochemical treatments generally altered PLA degradation more than chemical ones, except for the chemical NaOH treatment. Consequently, combining chemical NaOH treatment with electrochemical activation resulted in slight preservation of PLA compared to that of NaOH alone. Interestingly, greater PLA preservation was observed when chemical NaOH activation was combined with electrochemical NaOH treatment, surpassing even the results of the electrochemical NaOH treatment alone. The second degradation peak was much more influenced by the NaOH-EC_PBS_ combination, indicating that this combination facilitates the degradation of the polymer additive.

When chemical H_2_SO_4_ treatment was combined with electrochemical activation in NaOH, a slight increase in PLA decomposition temperature was observed without a pronounced change in *T*_*peak2*_. Combining H_2_SO_4_ with electrochemical PBS treatment resulted in a slight decrease in the PLA degradation temperature, while the polymer additive degradation peak shifted to a slightly higher temperature. Chemical treatment with DMF combined with electrochemical treatments resulted in a lower thermal stability of the electrodes, with *T*_*onset*_ and *T*_*peak1*_ considerably lower than those observed with DMF chemical treatment alone. The second degradation temperature was more affected by the combination of electrochemical PBS treatment.

After heating to 600 °C, all treatments resulted in approximately 19.8 ± 0.4% residue corresponding to CB, suggesting that the surface degradation induced by all tested treatments did not significantly alter the relative proportions of PLA and CB in the electrodes. These results indicate that while the treatments can modify the surface chemistry and morphology, they do not cause bulk compositional changes in the 3D-printed electrodes.

### 3.9 Dynamic Mechanical Analysis (DMA)

DMA was conducted to assess the effects of the surface post-treatments on the mechanical and viscoelastic properties of the electrodes. Representative DMA data are presented in Figure 5B–5E for the PLA filament, the CB/PLA filament, the untreated electrode, and the NaOH-EC_NaOH_-treated electrode, respectively. The thermal and mechanical properties extracted from the data are summarized in Table 5.

**Table 5:** Thermal and Mechanical Properties of the Samples

sample	*T*_*g*_ (°C)	tan δ peak height	tan δ peak area	*E′* at 30 °C (GPa)	*T*_*E*″_ (°C)
PLA filament	65.33	2.880	16.39	11.35	58.91
CB/PLA filament	62.73	1.100	5.98	9.27	56.39
No treatment	63.45	0.890	5.71	4.91	57.75
DMF	57.89	0.160	1.73	13.92	44.08
H_2_SO_4_	63.71	1.117	6.40	15.98	56.40
NaOH	64.34	1.065	6.23	21.61	57.41
EC_NaOH_	64.50	1.126	6.48	23.68	50.61
EQPBS	64.28	1.212	5.90	19.24	57.69
DMF-EC_NaOH_	56.99	0.196	0.80	16.79	45.48
DMF-EC_PBS_	57.34	0.177	0.60	19.10	54.03
H_2_SO_4_-EC_NaOH_	64.29	1.166	6.33	20.02	56.58
H_2_SO_4_-EC_PBS_	62.98	1.143	6.36	19.52	55.96
NaOH-EC_NaOH_	62.63	1.153	7.01	19.87	55.00
NaOH-EC_PBS_	63.66	1.114	6.02	15.49	56.46

All thermomechanical properties of the composite filament and 3D-printed electrodes were lower compared to pure PLA filament, likely due to the presence of CB, as high nanoparticle loadings (≥10 wt %) are known to reduce the molecular mobility of polymer chains.^84^ Typical CB particle sizes range from 10 to 500 nm.^85^

The *tan δ* peak area, which reflects the polymer chain participation in the glass transition,^86^ slightly decreased after electrode printing but increased for most treatments, except those involving DMF, which reduced both *tan δ* peak area and height. The tan δ peak height reflects the strength of interfacial interactions.^86^ Thus, the observed reductions suggest that DMF treatment, either alone or combined with electrochemical protocols, enhances interfacial interactions between polymer chains and CB, which is consistent with the observed reduction in glass transition temperature (*T*_g_).

The storage modulus (*E*′) measured at 30 °C (*E*′_30_) reflects the material stiffness at typical operating conditions, as the glass transition of PLA occurs around 60 °C.^87^ Except for the chemical DMF treatment (13.92 GPa), all treated electrodes exhibited *E’*_30_ values three to five times higher than the untreated electrode (4.91 GPa), indicating increased stiffness induced by the treatments. The EC_NaOH_ and NaOH treatments yielded the highest storage moduli (23.68 and 21.61 GPa, respectively). In contrast, the smallest increases in stiffness were observed for the NaOH-EC_PBS_ (15.49 GPa) and H_2_SO_4_ (15.98 GPa) treatments.

Importantly, no direct correlation was observed between electrochemical performance and stiffness (*E*′_30_), as electrodes from both high- and low-performing groups displayed varying stiffness values. This highlights the need for further investigation into the complex interplay among stiffness, flexibility, mechanical stability, and electrochemical performance in 3D-printed CB/PLA electrodes.^88^

## 4 Conclusions

In this work, a multiresponse statistical framework was developed to objectively compare the electrochemical performance of 3D-printed CB/PLA electrodes subjected to different surface activation protocols. Rather than proposing a new activation strategy or a novel electrode material, the primary contribution of this study is a reproducible methodology that integrates multiple performance indicators into a unified decision-making framework, addressing a common limitation in the literature, where activation protocols are typically compared using single electrochemical metrics, often without verifying whether the statistical assumptions underlying those comparisons (e.g., homogeneity of variance) actually hold, or without any rigorous statistical validation. By normalizing, weighting, and aggregating key electrochemical variables (*I*_*pa*_, |*I_pa_*/*I_pc_* |, Δ*E*_*p*_, and *R*_*ct*_) into a performance function (*L*) and a risk-adjusted lower-bound metric (*L*_99%_), the proposed approach enables objective ranking of activation protocols while explicitly accounting for experimental variability and reproducibility.

The proposed framework was demonstrated by using chemical, electrochemical, and combined activation treatments for 3D-printed CB/PLA electrodes. Among the evaluated protocols, alkaline treatments consistently exhibited the best overall performance. Although the combined NaOH-EC_NaOH_ protocol delivered the highest overall scores, the single-step NaOH treatment achieved comparable performance with a lower complexity and cost. These conditions provided high *L*_99%_ values and narrow dispersions, demonstrating reproducible and scalable activation routes for electroanalytical applications. Thus, both protocols effectively enhance the electrochemical performance of the 3D-printed electrodes.

Other characterizations supported these findings. CV and EIS revealed increased peak currents, reduced Δ*E*_*p*_, and markedly lower *R*_*ct*_ after alkaline activation; contact-angle measurements indicated greater exposure of the hydrophobic CB phase; and SEM-EDS evidenced surface modification with enhanced CB exposure. Electrical resistance decreased after several treatments, but conductivity alone did not predict top performance. ATR-FTIR analysis revealed a C = C band for the single-step NaOH treatment, likely attributed to the exposure of CB. Raman analysis revealed that induced disorder does not linearly correlate with electrochemical gains, emphasizing the need to balance defects with efficient electron-transfer pathways. TGA indicated no significant changes in bulk composition, while DMA revealed treatment-dependent mechanical responses.

Although the proposed framework proved to be effective for comparing activation protocols, the use of only three independent replicates per condition represents a limitation of this study. With only two degrees of freedom per treatment, the estimation of variance, and consequently the risk-adjusted score (*L*_99%_), is subject to uncertainty, and the assumption of normality underlying this score cannot be rigorously verified with such a small sample size. To partially mitigate this limitation, a conservative lower confidence bound was adopted by using *k* = 2.35 in the calculation of *L*_99%_, thereby penalizing treatments with a higher experimental variability. Nevertheless, future studies, including a larger number of independent replicates, will enable more reliable variance estimation and a more rigorous assessment of the underlying distributional assumptions.

In spite of these limitations, the use of triplicates was sufficient to provide reliable statistical interpretation and proper optimization of the experimental results, clearly demonstrating the robustness and appropriateness of the proposed framework. Furthermore, the *L*/*L*_99%_ framework approach is general and readily transferable to other research areas in which multiple variables must be analyzed simultaneously (e.g., development of conductive composites and inks, materials for energy and environmental applications, development of (bio)­sensing and catalytic platforms, nanomaterial tuning, bioreceptor immobilization, and development of targeted drug and gene delivery systems, among others). Additionally, it can be applied to optimize various surface post-treatments, such as NaOH concentration and time, via experimental design. By applying response surface modeling of the *L*_99%_ values, it is possible to identify activation conditions that outperform the 3 mol L^–1^ for 30 min protocol, ensuring maximum performance while preserving the simplicity, reproducibility, and scalability of the process. Overall, this study provides not only high-performing and reproducible alkaline activation protocols for 3D-printed CB/PLA electrodes but also a statistical and reproducible methodology to guide multivariate and multivariable analyses across different fields.

## Supplementary Material





## Notes

Raw Data (Electrochemical Characterization) (XLSX)
Images of the CB/PLA electrodes; cyclic voltammograms used to select the chemical and electrochemical treatment conditions; equivalent circuits fitted to the EIS data; electrochemical characterization of untreated and surface-treated electrodes; electroactive area determined by nonlinear regression; Levene’s test assessing the homogeneity of variances of electrochemical variables for untreated and surface-treated electrodes; electrical resistance determined by linear regression; SEM images of the filament and untreated/surface-treated electrodes; chemical structures of PLA and CB; and a worksheet containing the raw electrochemical characterization data for both untreated and treated electrodes (electrode, scan rate, *I*_*pa*_, |*I_pa_*/*I_pc_* |, Δ*E*_*p*_, and *R*_*ct*_) (PDF)

## Abbreviations

3D: three-dimensional
AC: alternating current
ANOVA: analysis of variance
ATR-FTIR: attenuated total reflection Fourier transform infrared spectroscopy
CA: chronoamperometry
CB: carbon black
CV: cyclic voltammetry
DMA: dynamic mechanical analysis
DMF: N,N-dimethylformamide
EIS: electrochemical impedance spectroscopy
ESG: environmental, social, and governance
FDM: fused deposition modeling
FWHM: full width at half-maximum
LSV: linear sweep voltammetry
OCP: open-circuit potential
OLS: ordinary least-squares
PBS: phosphate-buffered saline
PLA: polylactic acid
SEM-EDS: scanning electron microscopy with energy-dispersive X-ray spectroscopy
TGA: thermogravimetric analysis
WLS: weighted least-squares.
