# Supplementary material for: Statistical Framework for Multiresponse Analyses: Comparative Assessment of Surface Activation Strategies for 3D-Printed Carbon Black/Polylactic Acid Electrodes toward Electrochemical (Bio)Sensing Applications
Source: ACS Omega. 2026 Sep 25;11(39):59490–511. doi: 10.1021/acsomega.6c05769 (PMC13647375; doi:10.1021/acsomega.6c05769)
Supplement: Supplementary file 1 [file ao6c05769_si_002.pdf]

Supplementary Material

A Statistical Framework for Multiresponse Analyses: Comparative Assessment of Surface Activation Strategies for 3D-Printed Carbon Black/Polylactic Acid Electrodes toward Electrochemical (Bio)Sensing Applications

Leonan dos Santos Rodrigues[a*], *Guilherme Sales da Rocha*[a], *Franccesca Fornasier*[a], *Andresa Viana Ramos*[b], *Elton Jorge da Rocha Rodrigues*[c], *José Carlos Costa da Silva Pinto*[a], *João Victor Nicolini*[d], *and Helen Conceição Ferraz*[a].

[a]Chemical Engineering Program, Alberto Luiz Coimbra Institute for Graduate Studies and Research in Engineering (Coppe), Federal University of Rio de Janeiro, 21941-914, Rio de Janeiro-RJ, Brazil

[b]Nanotechnology Engineering Program, Alberto Luiz Coimbra Institute for Graduate Studies and Research in Engineering (Coppe), Federal University of Rio de Janeiro, 21941-972, Rio de Janeiro-RJ, Brazil

[c] Professor Eloisa Mano Macromolecules Institute (IMA), Federal University of Rio de Janeiro, 21941-598, Rio de Janeiro-RJ, Brazil

[d] Graduate Program in Materials Science and Engineering, Institute of Technology, Federal Rural University of Rio de Janeiro, 23897-000, Seropédica-RJ, Brazil

*Email: leonan@peq.coppe.ufrj.br

Carbon black/polylactic acid electrodes

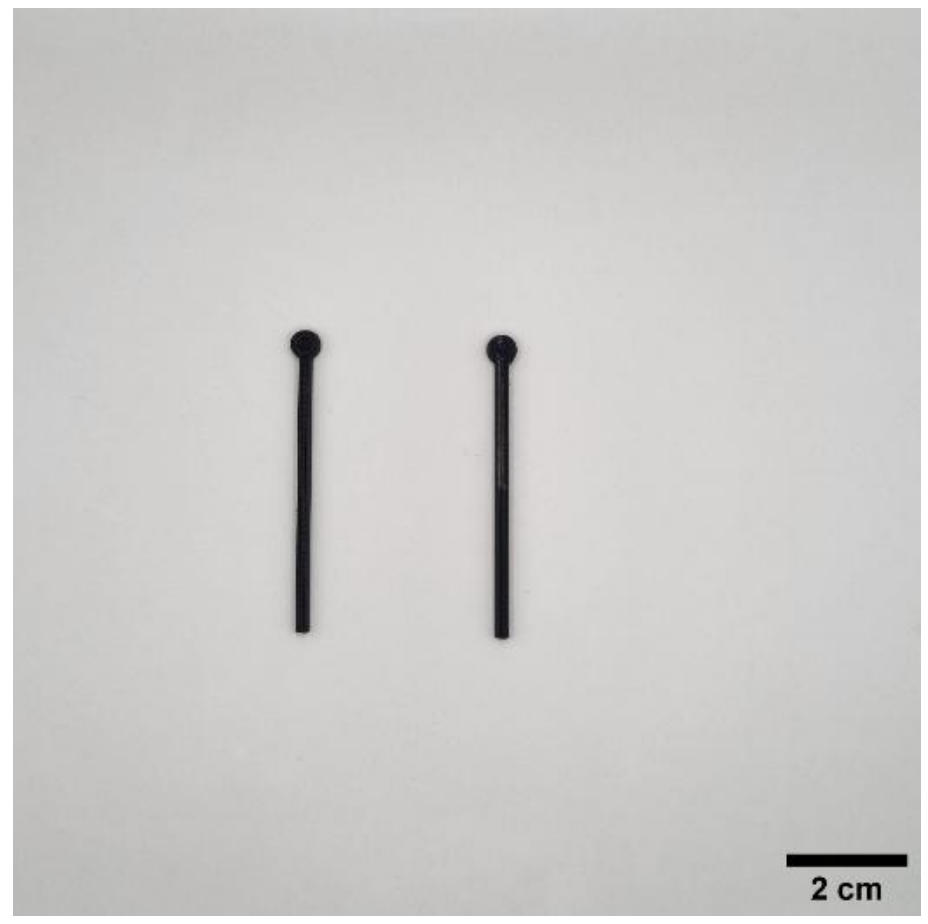

Figure S1. Pendulum-shaped electrodes (52.5 × 5 × 2 mm) fabricated using a fused deposition modeling (FDM) 3D printer (Ender-3S1 Plus, Creality, China).

Selection of the conditions under which the treatments were performed

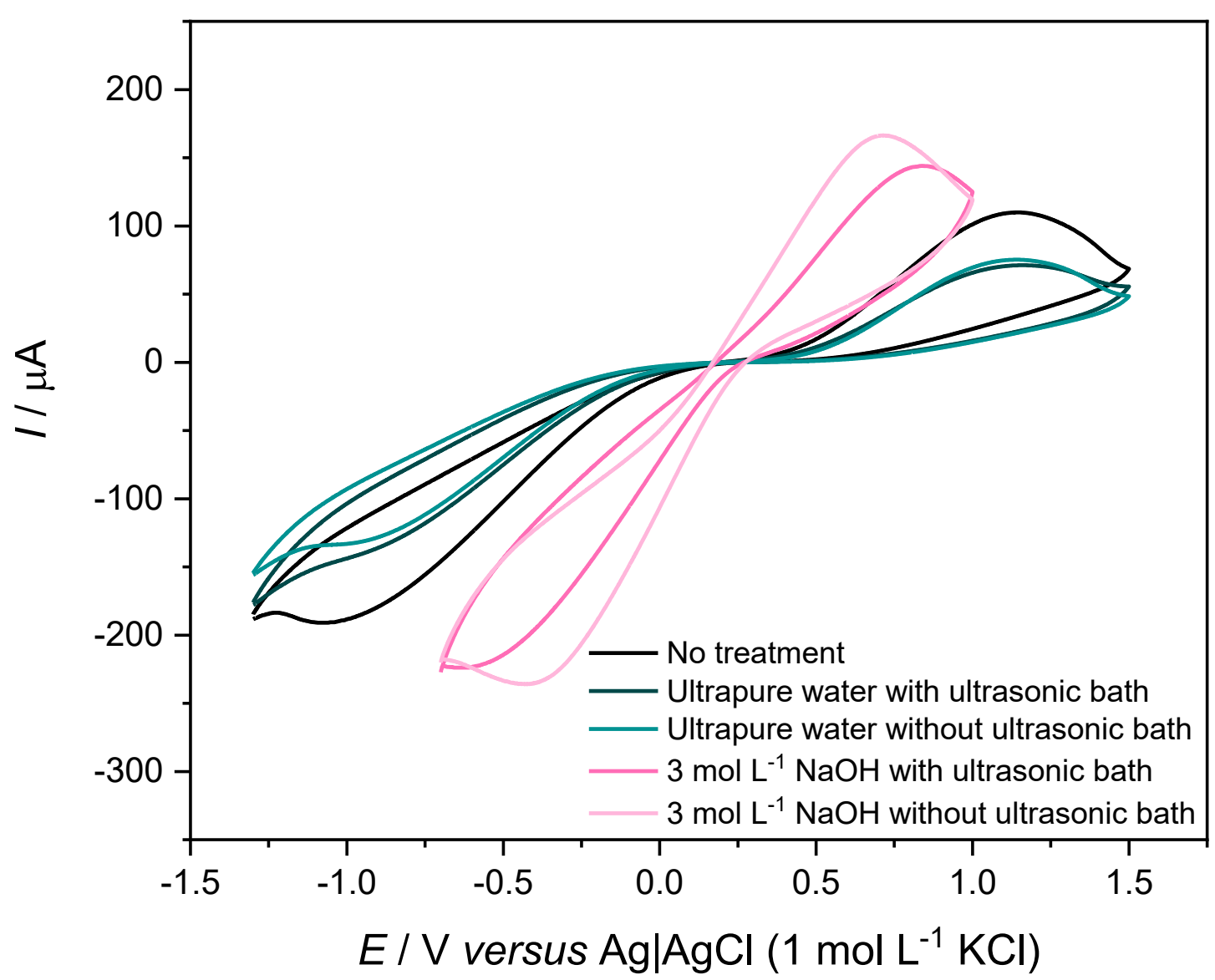

Figure S2. Cyclic voltammograms used to select the conditions for performing the chemical treatments with or without ultrasonic bath (40 kHz working frequency). All experiments were performed in a 5 mmol L^{-1} $K_3Fe(CN)_6$ solution containing 0.1 mol L^{-1} KCl (50 mV s^{-1}).

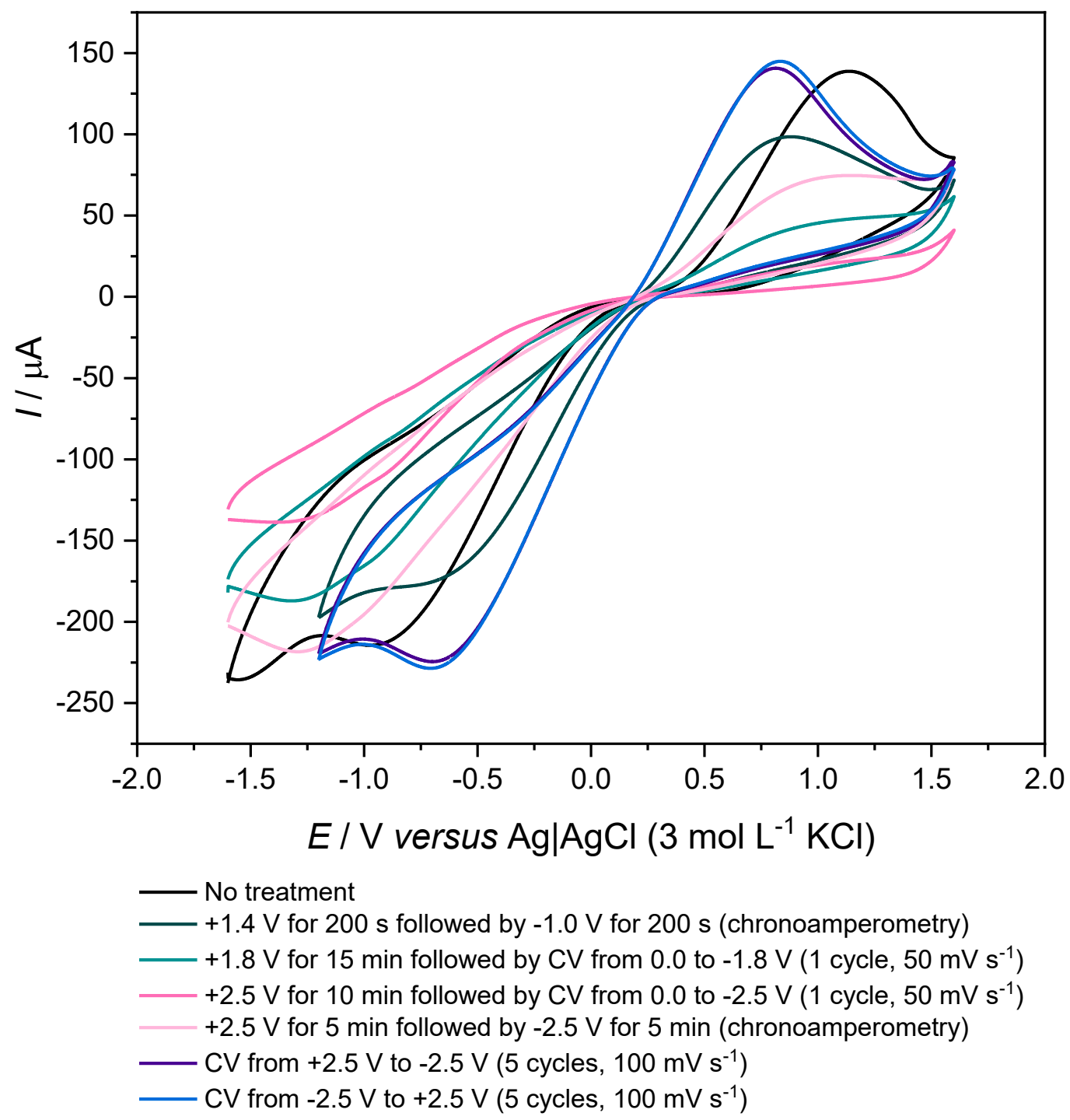

Figure S3. Cyclic voltammograms used to select the conditions for performing the electrochemical activation. All experiments were performed in a 5 mmol L^{-1} $K_3Fe(CN)_6$ solution containing 0.1 mol L^{-1} KCl (50 mV s^{-1}). Activation was conducted in 0.1 mol L^{-1} NaOH.

Equivalent circuits fitted to the EIS data

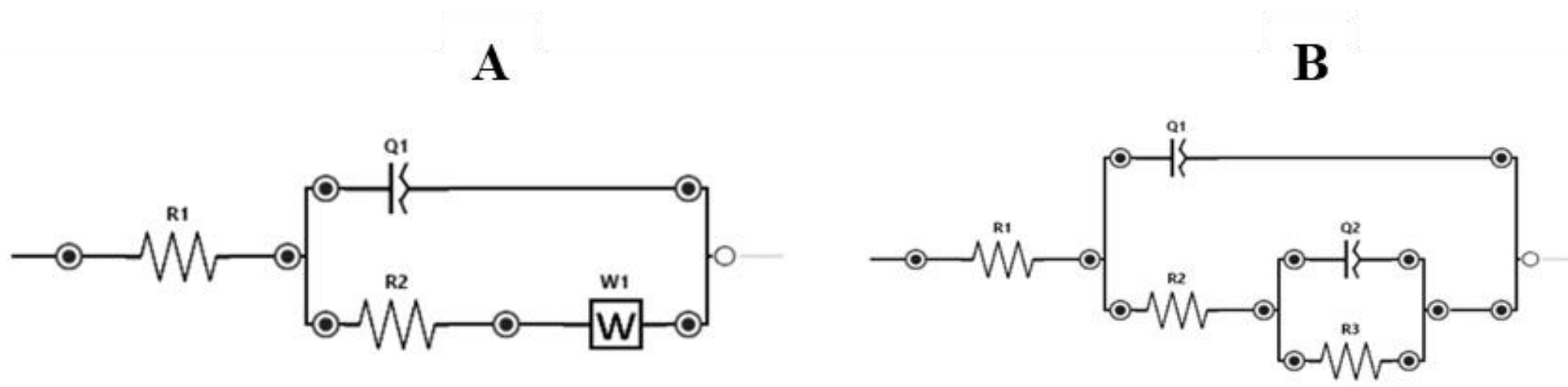

Figure S4. (A) Randles equivalent circuit used to fit the results for all electrodes, except for the electrode treated with DMF-EC_{PBS}. R1: solution resistance (R_s), R2: charge transfer resistance (R_{ct}), Q1: constant phase element (representing the double-layer capacitance, C_{dl}), and W1: Warburg element (W).

(B) Circuit used to fit the data obtained from electrodes treated with DMF-EC_{PBS}. R1: solution resistance (R_s), R2: additional resistance, likely due to pores formed by surface deformations caused by the treatment (R_{po}), Q1: additional constant phase element, possibly related to the charge accumulation within those pores by charged species (C_c), R3: charge transfer resistance (R_{ct}), and Q2: constant phase element (representing the double-layer capacitance, C_{dl}).

The capacitor was replaced by a constant phase element (CPE), allowing for a better fit.

Electrochemical characterization

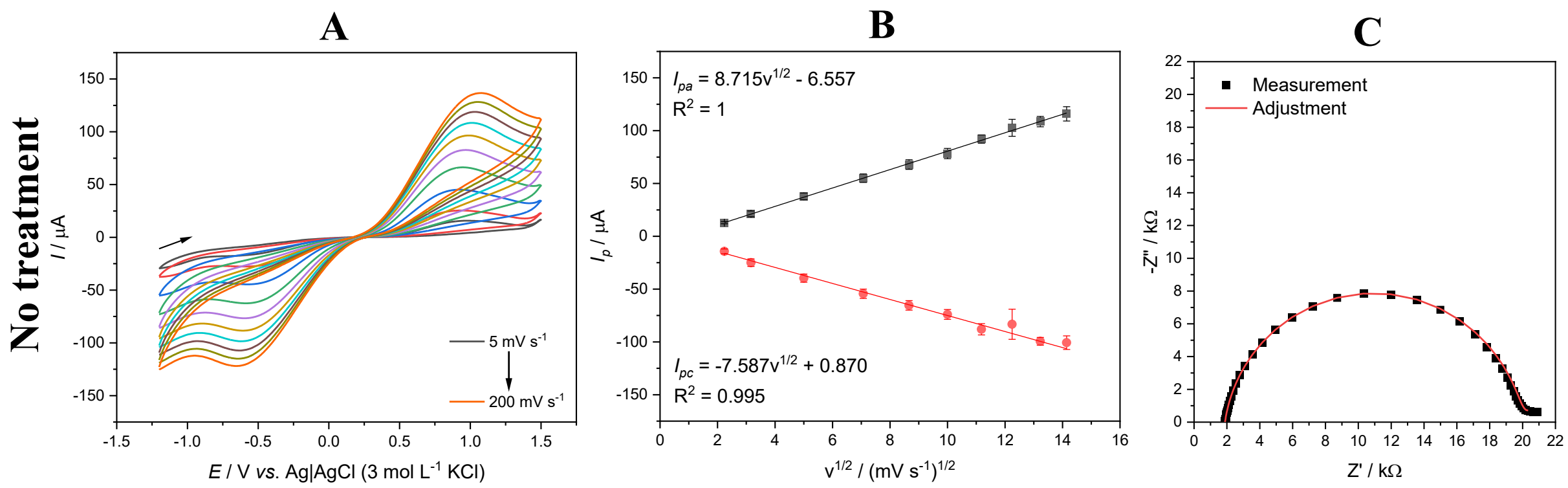

Figure S5. Cyclic voltammograms (5–200 mV s^{-1}) *vs.* Ag|AgCl (3 mol L^{-1} KCl) (A), I_{pa} and I_{pc} $vs.$ $v^{1/2}$ (B), and Nyquist plot (C) of the untreated electrodes. All experiments were performed in 1:1 mmol L^{-1} $[Fe(CN)_6]^{3-/4-}$ in 0.1 mol L^{-1} KCl.

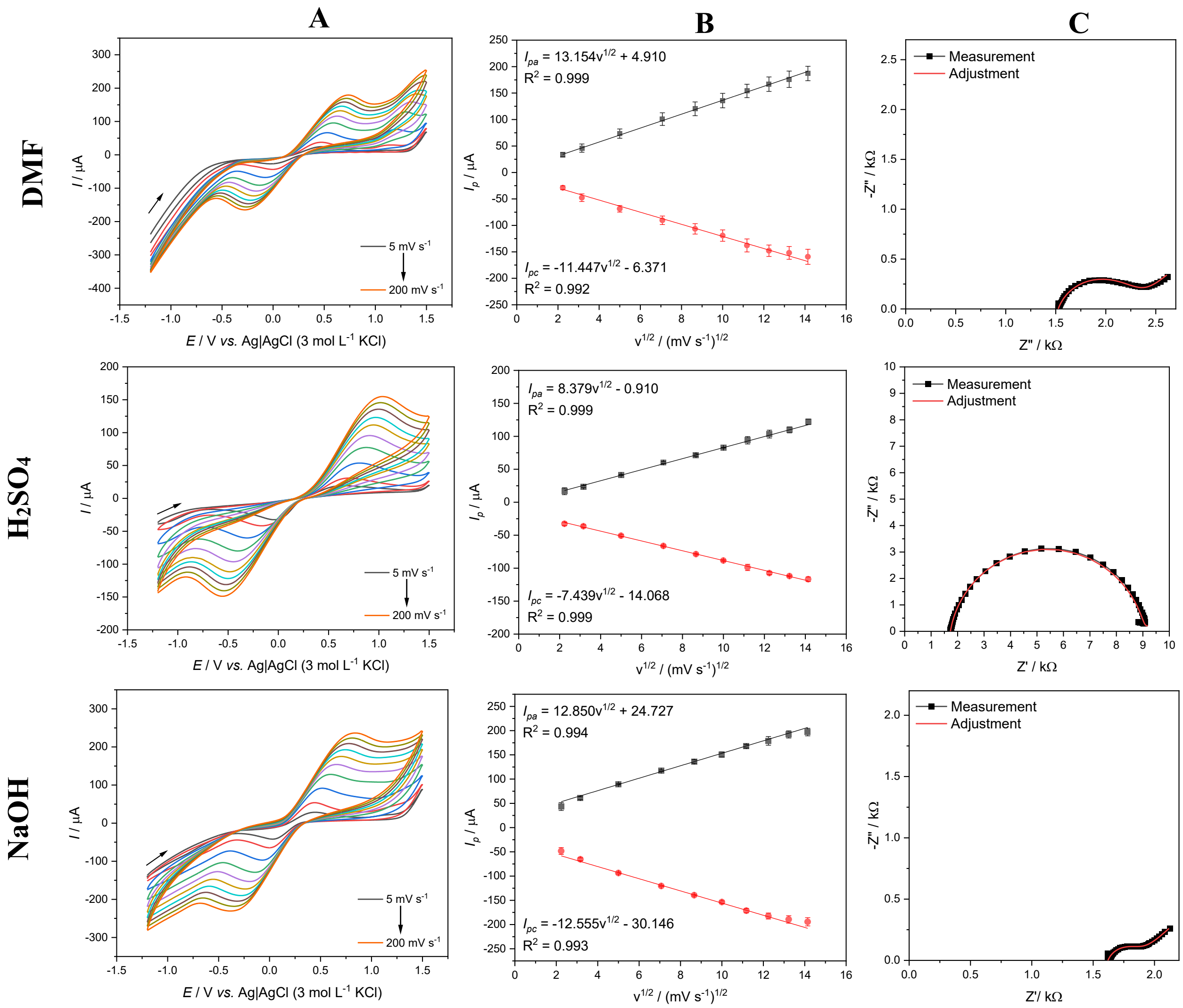

Figure S6. Cyclic voltammograms (5–200 mV s^{-1}) *vs.* Ag|AgCl (3 mol L^{-1} KCl) (A), I_{pa} and I_{pc} *vs.* $v^{1/2}$ (B), and Nyquist plot (C) of the electrodes chemically treated in DMF, 1 mol L^{-1} H_2SO_4, and 3 mol L^{-1} NaOH. All experiments were performed in 1:1 mmol L^{-1} $[Fe(CN)_6]^{3-/4-}$ in 0.1 mol L^{-1} KCl.

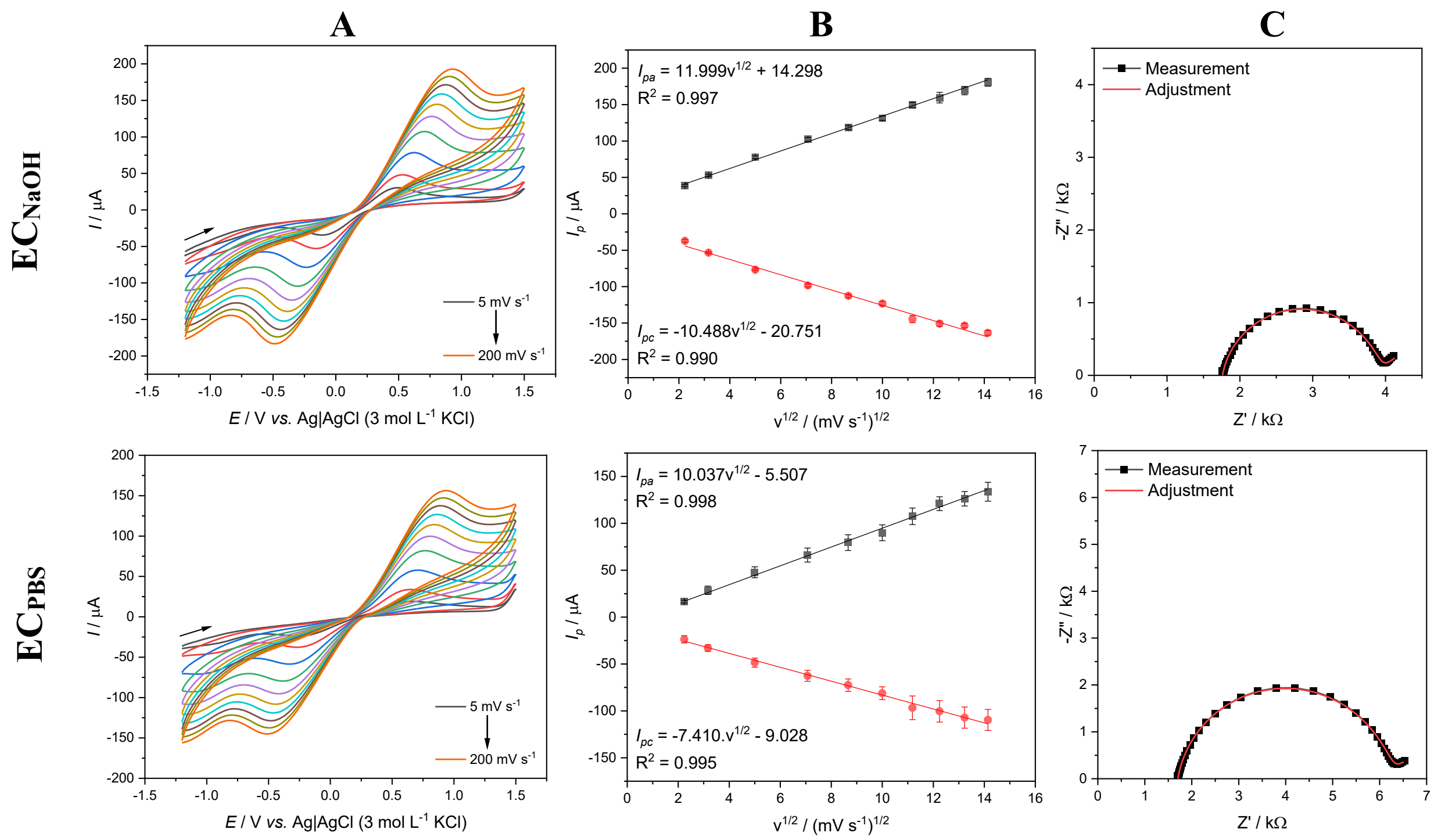

Figure S7. Cyclic voltammograms (5–200 mV s^{-1}) *vs.* Ag|AgCl (3 mol L^{-1} KCl) (A), I_{pa} and I_{pc} $vs.$ $v^{1/2}$ (B), and Nyquist plot (C) of the electrodes electrochemically treated with 0.1 mol L^{-1} NaOH (EC_{NaOH}) and 0.1 mol L^{-1} PBS (EC_{PBS}). All experiments were performed in 1:1 mmol L^{-1} $[Fe(CN)_6]^{3-/4-}$ in 0.1 mol L^{-1} KCl.

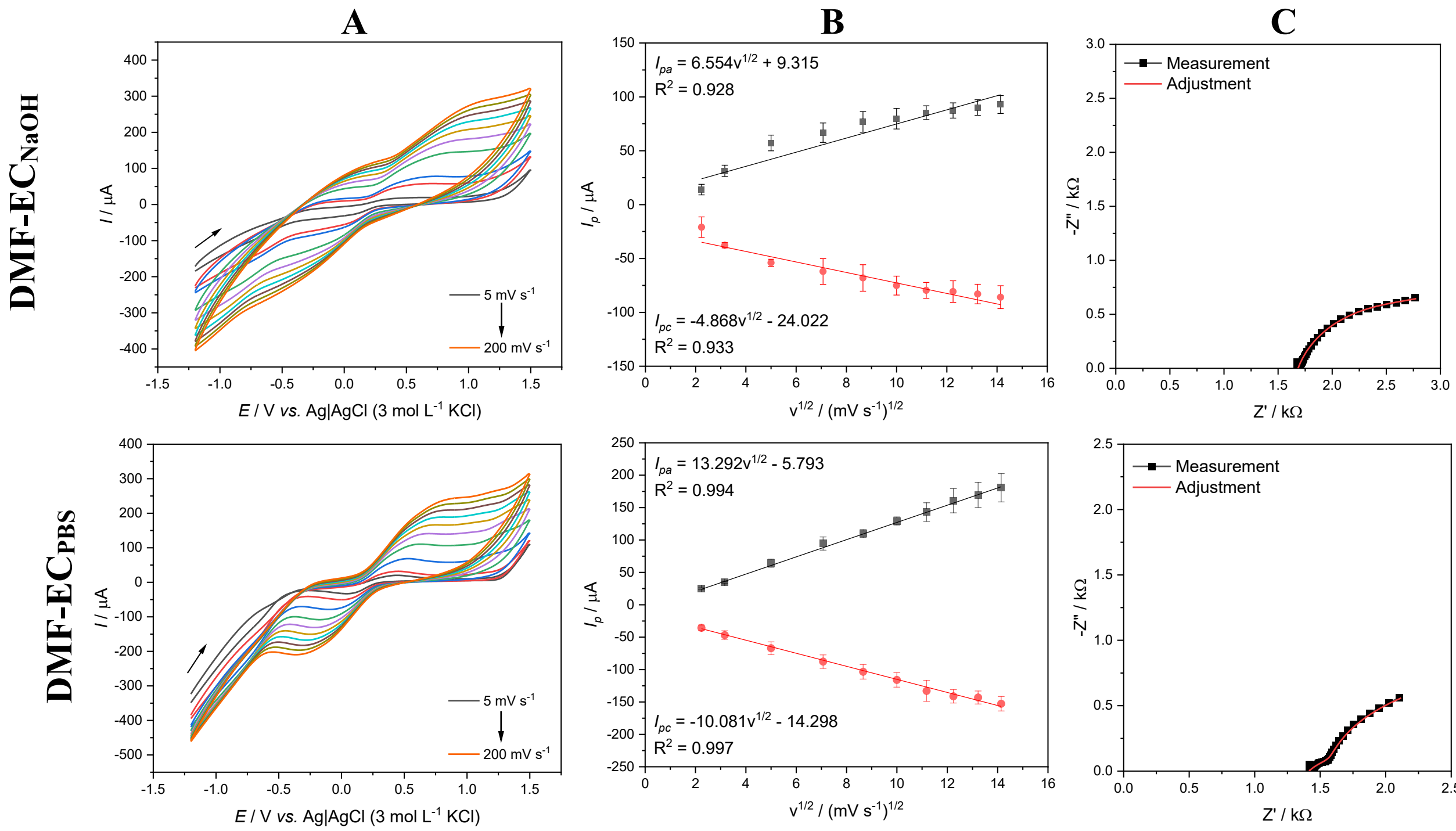

Figure S8. Cyclic voltammograms (5–200 mV s^{-1}) *vs.* Ag|AgCl (3 mol L^{-1} KCl) (A), I_{pa} and I_{pc} *vs*. $v^{1/2}$ (B), and Nyquist plot (C) of the electrodes chemically treated with DMF followed by electrochemical activation in 0.1 mol L^{-1} NaOH or 0.1 mol L^{-1} PBS. All experiments were performed in 1:1 mmol L^{-1} $[Fe(CN)_6]^{3-/4-}$ in 0.1 mol L^{-1} KCl.

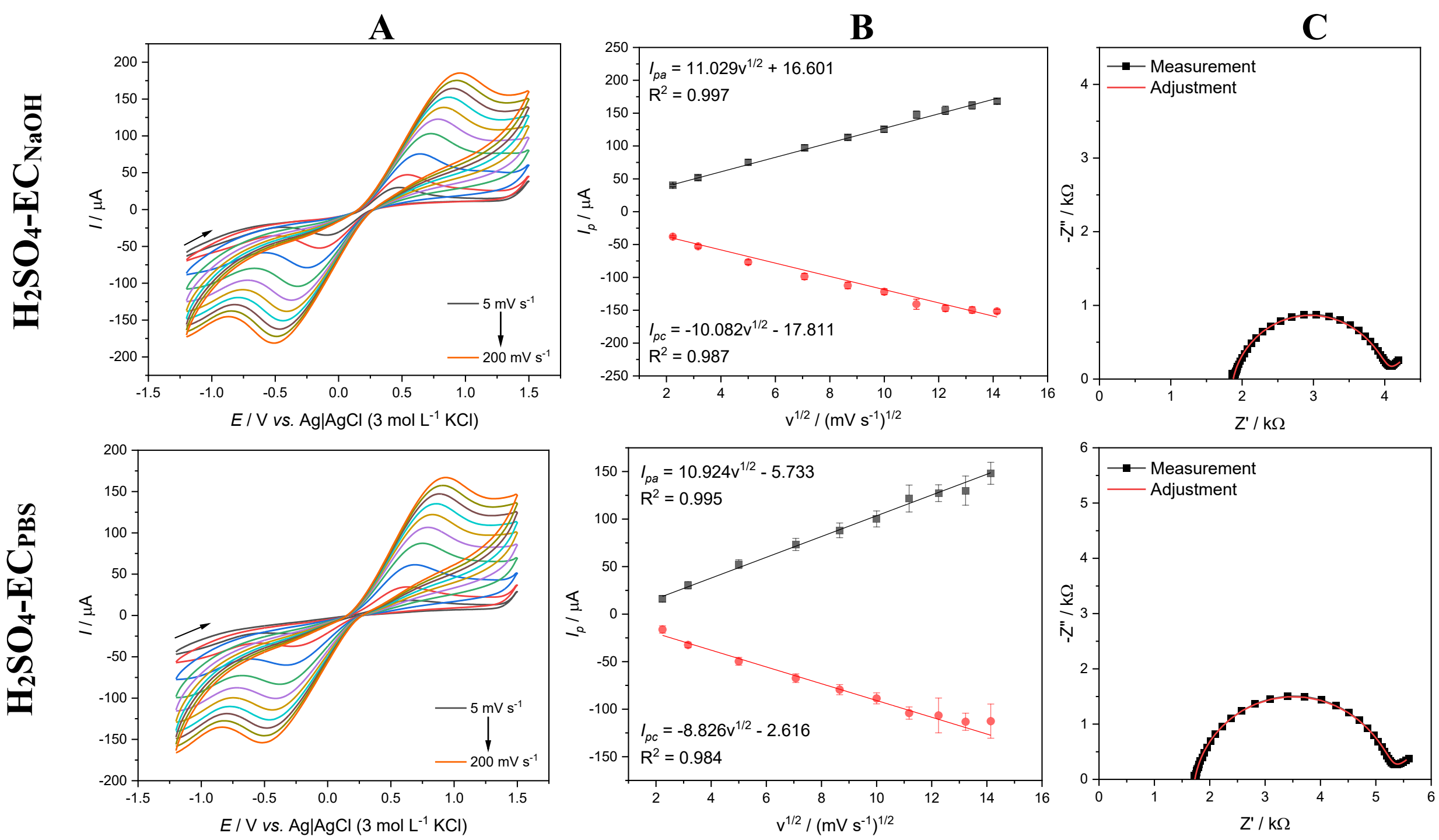

Figure S9. Cyclic voltammograms (5–200 mV s^{-1}) *vs.* Ag|AgCl (3 mol L^{-1} KCl) (A), I_{pa} and I_{pc} *vs*. $v^{1/2}$ (B), and Nyquist plot (C) of the electrodes chemically treated with 1 mol L^{-1} H_2SO_4 followed by electrochemical activation in 0.1 mol L^{-1} NaOH or 0.1 mol L^{-1} PBS. All experiments were performed in 1:1 mmol L^{-1} $[Fe(CN)_6]^{3-/4-}$ in 0.1 mol L^{-1} KCl.

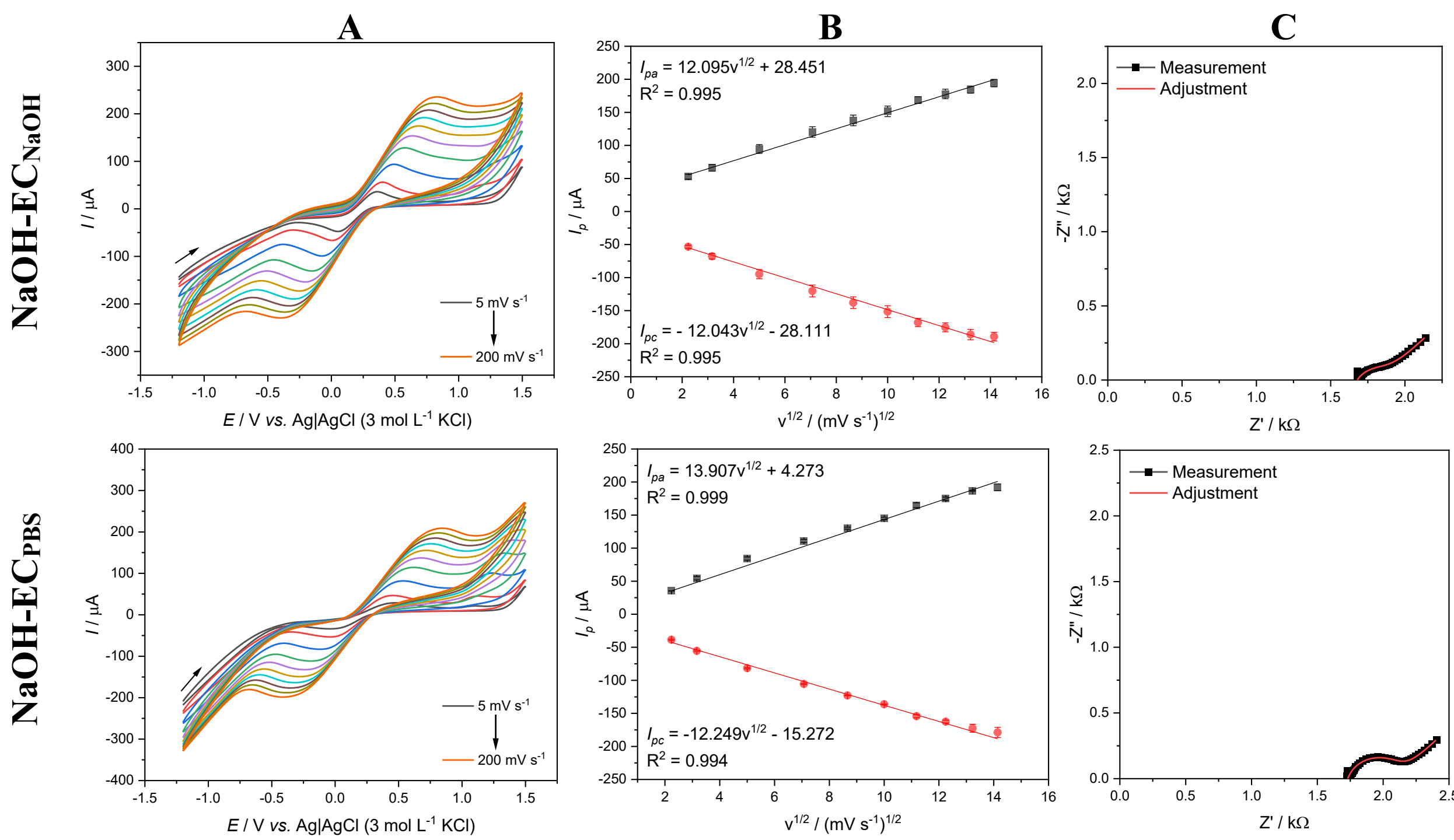

Figure S10. Cyclic voltammograms (5–200 mV s^{-1}) *vs.* Ag|AgCl (3 mol L^{-1} KCl) (A), I_{pa} and I_{pc} *vs.* $v^{1/2}$ (B), and Nyquist plot (C) of the electrodes chemically treated with 3 mol L^{-1} NaOH followed by electrochemical activation in 0.1 mol L^{-1} NaOH or 0.1 mol L^{-1} PBS. All experiments were performed in 1:1 mmol L^{-1} $[Fe(CN)_6]^{3-/4-}$ in 0.1 mol L^{-1} KCl.

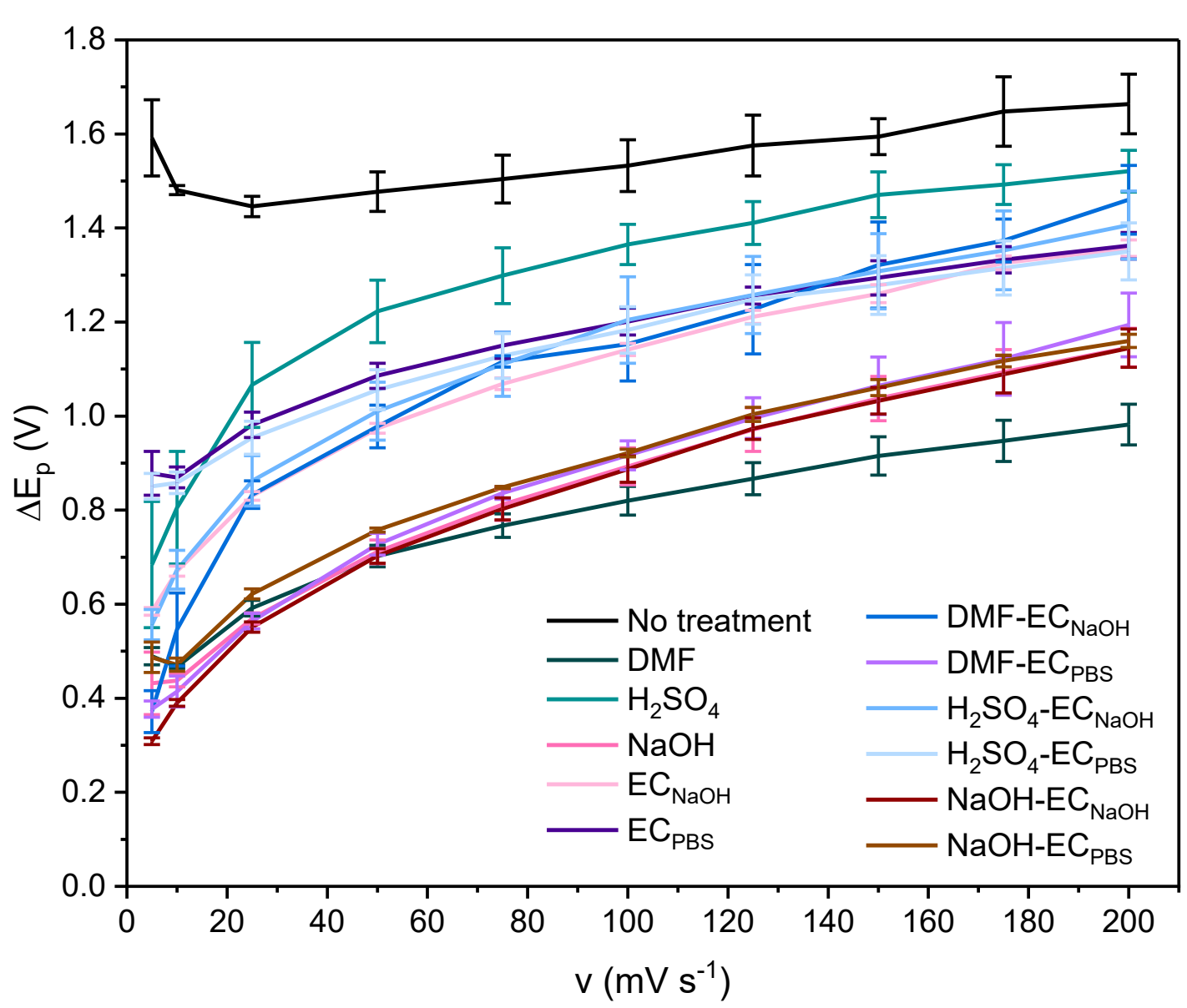

Figure S11. Peak-to-peak potential separation (ΔE_p) on scan rate (v) for 3D-printed electrodes subjected to different activation treatments. All experiments were performed in 1:1 mmol L^{-1} $[Fe(CN)_6]^{3-/4-}$ in 0.1 mol L^{-1} KCl.

Electroactive area

Table S1. Electroactive area for 3D-printed electrodes subjected to different surface post-treatments.

Treatment	Electroactive area / cm^2<br>Model: Ipa=269000*Sqrt(0.0000067)*0.000001*Ae*Sqrt(v)<br>Loss function: $(OBS-PRED)^2$ (homogeneous variance)<br>$(OBS-PRED)^2/\sigma^2_{Ipa}$ (heterogeneous variance)<br>Level of confidence: 95.0%								
	**Electrode #1**			**Electrode #2**			**Electrode #3**		
	**Estimate**	**Standard error**	***R***	**Estimate**	**Standard error**	***R***	**Estimate**	**Standard error**	***R***
No treatment	0.35335	0.00615	0.99360	0.35798	0.00399	0.99714	0.39242	0.00503	0.99627
DMF	0.68095	0.00961	0.99392	0.5931	0.0043	0.99858	0.5785	0.0038	0.99885
H_2SO_4	0.3770	0.0021	0.99483	0.3757	0.0015	0.99593	0.3753	0.0022	0.99185
NaOH	0.65494	0.02072	0.95893	0.69308	0.01364	0.98913	0.68595	0.01286	0.98677
EC_{NaOH}	0.59793	0.01242	0.98542	0.58665	0.01164	0.98663	0.62017	0.01083	0.99010
EC_{PBS}	0.39803	0.00599	0.99475	0.4654	0.0042	0.99789	0.42752	0.00511	0.99337
DMF-EC_{NaOH}	0.31558	0.01381	0.93790	0.33141	0.01736	0.89154	0.37730	0.01958	0.88635
DMF-EC_{PBS}	0.51434	0.01040	0.99559	0.54959	0.02033	0.94906	0.53701	0.01502	0.97037
H_2SO_4- EC_{NaOH}	0.58727	0.01584	0.97350	0.58699	0.01283	0.98345	0.55376	0.01108	0.98666
H_2SO_4- EC_{PBS}	0.31310	0.01359	0.93767	0.33100	0.01768	0.89152	0.37437	0.01912	0.88605
NaOH- EC_{NaOH}	0.65326	0.01926	0.96576	0.66740	0.01985	0.96502	0.70588	0.02657	0.93644
NaOH- EC_{PBS}	0.68417	0.01337	0.97584	0.68355	0.01226	0.98434	0.68532	0.01170	0.98808

R: correlation coefficient between experimental and model-predicted values obtained from nonlinear regression.

Selection of promising treatments

Table S2. Levene's test assessing the homogeneity of variances for anodic peak current (I_{pa}), anodic-to-cathodic peak current ratio ($|I_{pa}/I_{pc}|$), and peak-to-peak potential separation (ΔE_p) across different scan rates (5–200 mV s^{-1}) within each electrode treatment. A p-value < 0.05 was considered indicative of statistically significant heterogeneity of variances.

Treatment	I_{pa} (A)		$\lvert I_{pa}/I_{pc}\rvert$		ΔE_p (V)	
	F	***p***	***F***	***p***	***F***	***p***
No treatment	1.872865	0.116315	6.765557	0.000192	1.001037	0.470387
DMF	1.141862	0.380645	2.069000	0.084332	1.099145	0.406350
H_2SO_4	2.533975	0.039978	6.765557	0.000192	1.841007	0.122577
NaOH	2.155306	0.073280	3.085829	0.017167	1.930355	0.105825
EC_{NaOH}	2.117340	0.077944	4.131471	0.003959	0.375047	0.933678
EC_{PBS}	0.692601	0.707836	4.649649	0.002036	0.699438	0.702268
DMF-EC_{NaOH}	0.471890	0.876474	2.264058	0.061461	1.162899	0.368475
DMF-EC_{PBS}	2.981497	0.020067	1.766578	0.138569	1.557887	0.195377
H_2SO_4- EC_{NaOH}	0.642847	0.748171	5.335646	0.000894	0.908849	0.536564
H_2SO_4- EC_{PBS}	2.509165	0.041573	3.424954	0.010464	0.488661	0.865122
NaOH- EC_{NaOH}	0.574907	0.801869	1.804381	0.130199	3.016266	0.019046
NaOH- EC_{PBS}	2.567834	0.037906	1.822366	0.126398	3.290641	0.012702

Electrical Resistance

Table S3. Electrical resistance determined from E versus I linear fits for 3D-printed electrodes subjected to different surface post-treatments.

**Treatment**	**Electrical Resistance / MΩ**<br>Linear fit: $E = RI$<br>Loss function: (OBS-PRED)^2<br>Level of confidence: 95.0%								
	**Electrode #1**			**Electrode #2**			**Electrode #3**		
	**Estimate**	**Standard error**	***R***	**Estimate**	**Standard error**	***R***	**Estimate**	**Standard error**	***R***
No treatment	0.00208	6.64696E-7	0.9999	0.00212	5.45144E-7	0.99993	0.0022	6.56857E-7	0.99991
DMF	0.00158	2.9228E-7	0.99997	0.00153	2.51902E-7	0.99997	0.00159	1.58508E-7	0.99999
H_2SO_4	0.00206	4.82986E-7	0.99995	0.00201	6.82955E-7	0.99988	0.0026	6.43383E-7	0.99994
NaOH	0.00174	3.86773E-7	0.99995	0.00173	2.87449E-7	0.99997	0.00184	3.88072E-7	0.99996
EC_{NaOH}	0.00237	9.54317E-7	0.99984	0.00208	7.24207E-7	0.99988	0.00196	1.66408E-7	0.99999
EC_{PBS}	0.00257	1.64735E-6	0.99959	0.0022	5.99086E-7	0.99993	0.00253	5.68234E-7	0.99995
DMF-EC_{NaOH}	0.00185	2.90603E-7	0.99998	0.00186	2.56112E-7	0.99998	0.00182	4.50264E-7	0.99994
DMF-EC_{PBS}	0.00146	2.24265E-7	0.99998	0.00151	2.47809E-7	0.99997	0.0016	2.66094E-7	0.99997
H_2SO_4- EC_{NaOH}	0.00219	7.85898E-7	0.99987	0.00281	1.11966E-6	0.99984	0.00224	7.91202E-7	0.99988
H_2SO_4- EC_{PBS}	0.0023	1.10231E-6	0.99977	0.00195	2.02844E-7	0.99999	0.00222	7.58632E-7	0.99988
NaOH- EC_{NaOH}	0.00194	5.03457E-7	0.99993	0.0019	3.81944E-7	0.99996	0.00189	4.43291E-7	0.99994
NaOH- EC_{PBS}	0.00198	5.03066E-7	0.99994	0.00195	5.74844E-7	0.99991	0.00202	3.17691E-7	0.99998

R: correlation coefficient between experimental and model-predicted values obtained from linear regression.

SEM images

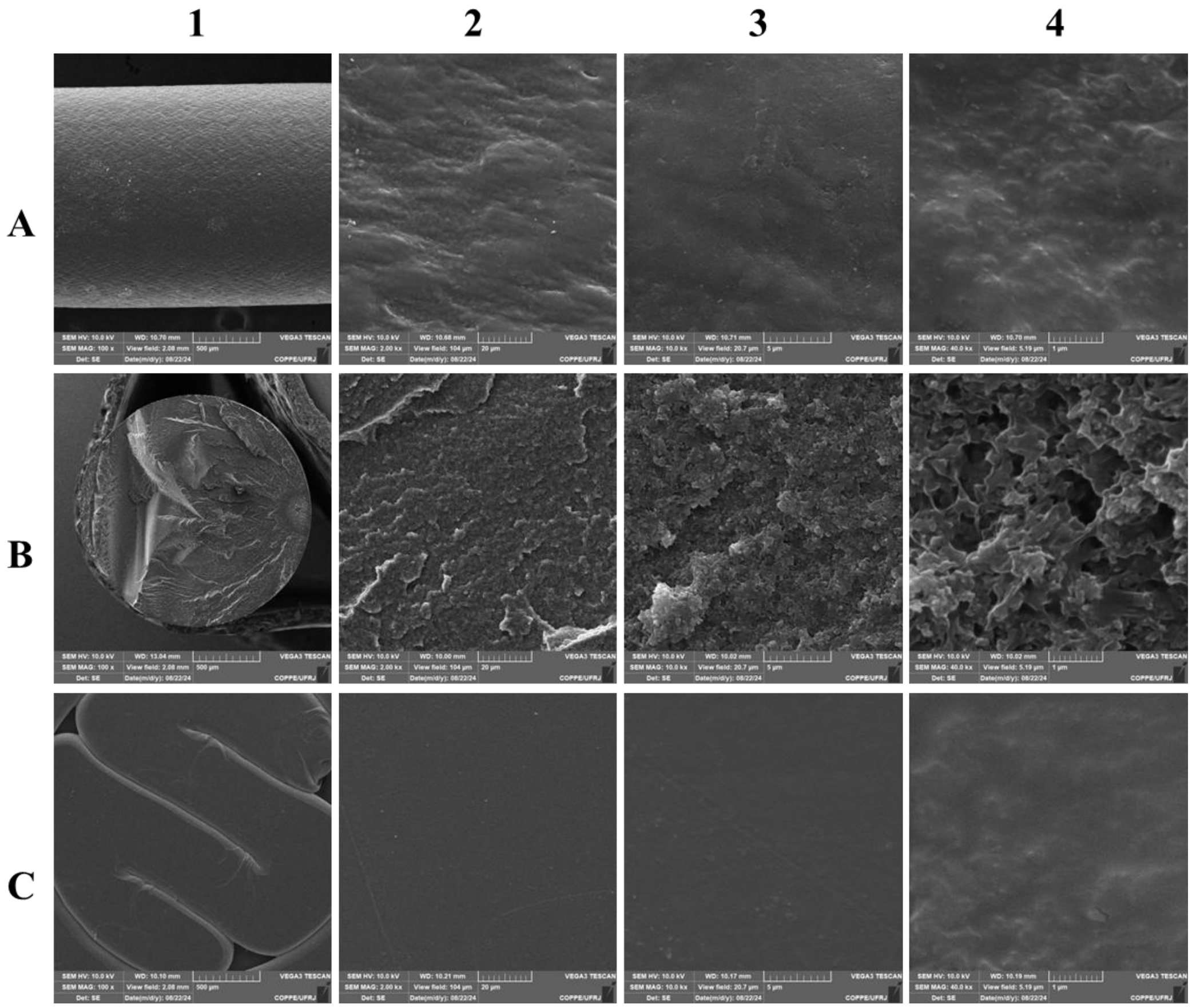

Figure S12. SEM images of the filament (CB/PLA) (A), the CB/PLA cross section (B), and the untreated electrode (C). The magnifications used were 100× (1), 2,000× (2), 10,000× (3), and 40,000× (4).

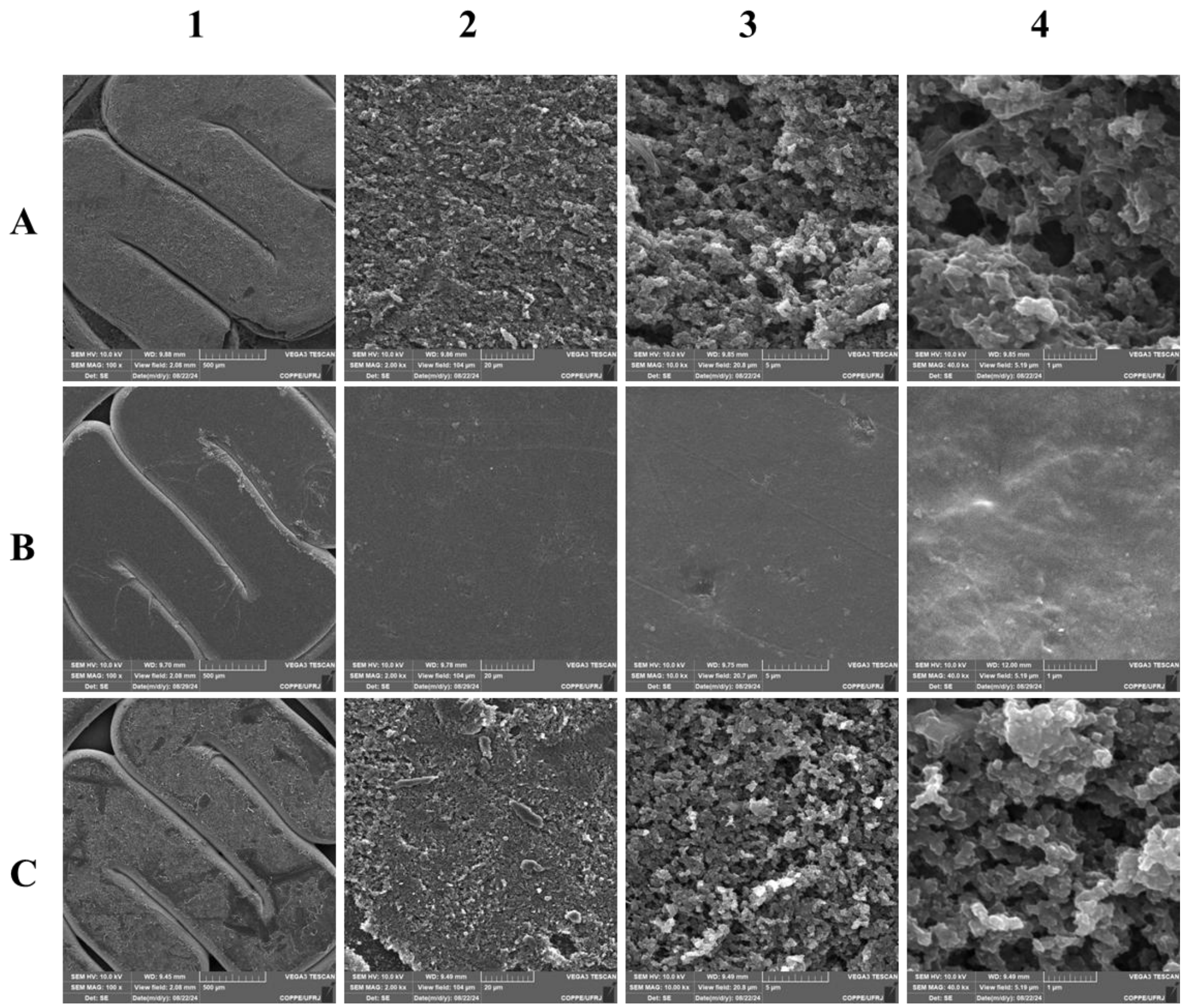

Figure S13. SEM images of the electrodes chemically treated with DMF (A), H_2SO_4 (B), and NaOH (C). The magnifications used were 100× (1), 2,000× (2), 10,000× (3), and 40,000× (4).

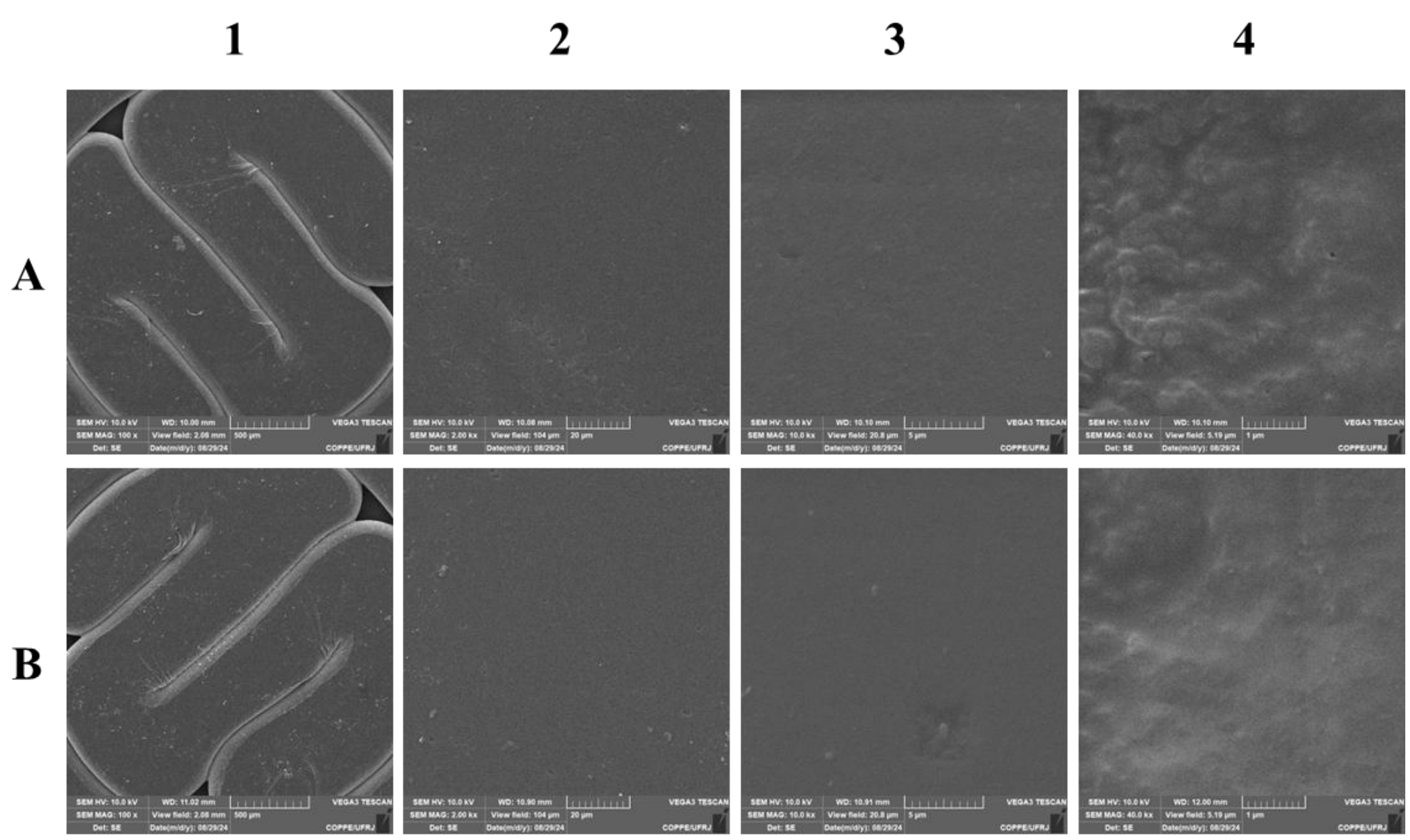

Figure S14. SEM images of the electrodes electrochemically treated: EC_{NaOH} (A) and EC_{PBS} (B). The magnifications used were 100× (1), 2,000× (2), 10,000× (3), and 40,000× (4).

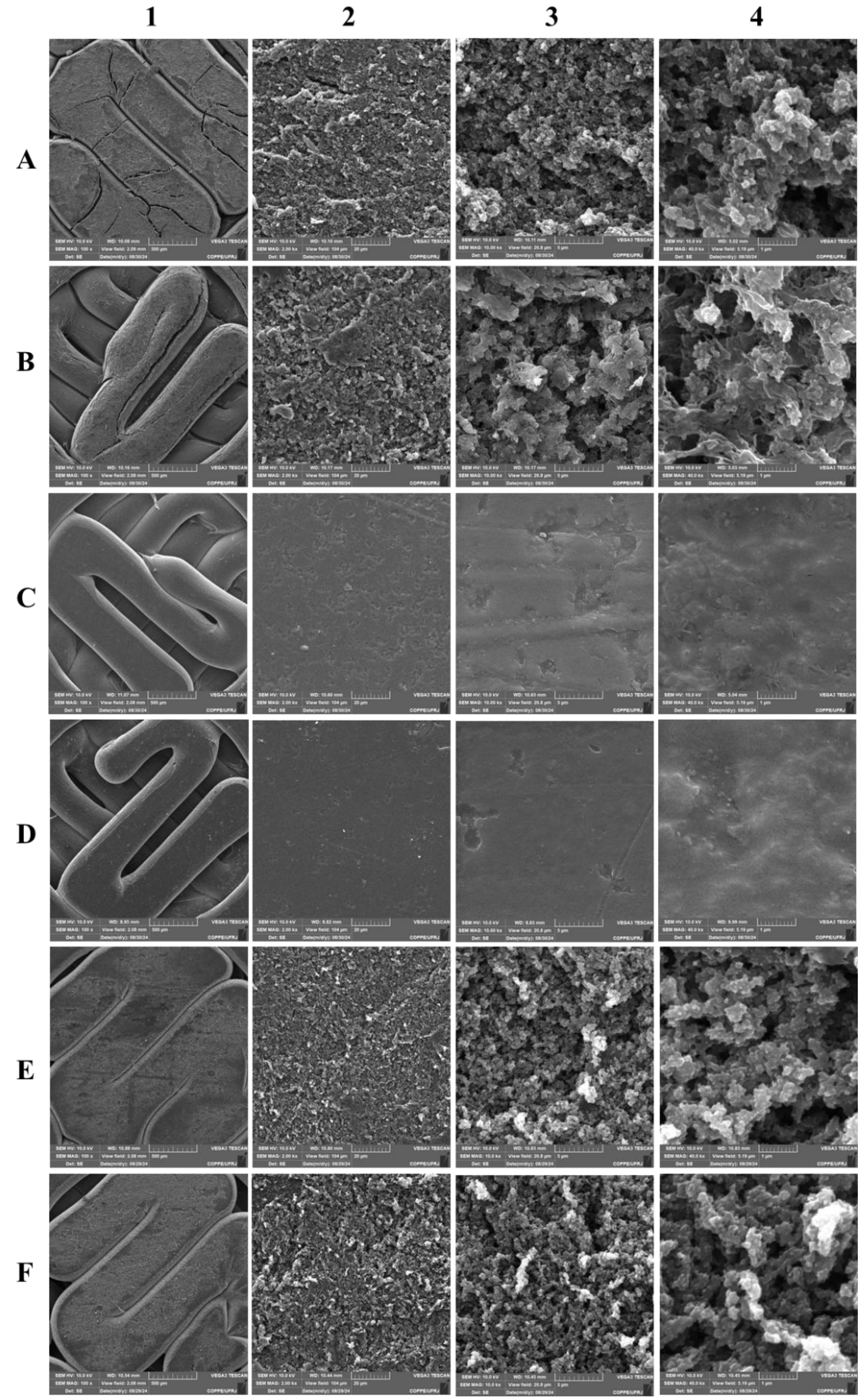

Figure S15. SEM images of the electrodes subjected to the combined treatments: DMF-EC_{NaOH} (A), DMF-EC_{PBS} (B), H_2SO_4-EC_{NaOH} (C), H_2SO_4-EC_{PBS} (D), NaOH-EC_{NaOH} (E), NaOH-EC_{PBS} (F). The magnifications used were 100× (1), 2,000× (2), 10,000× (3), and 40,000× (4).

Polylactic acid (PLA) and carbon black (CB) structures

(A)

(B)

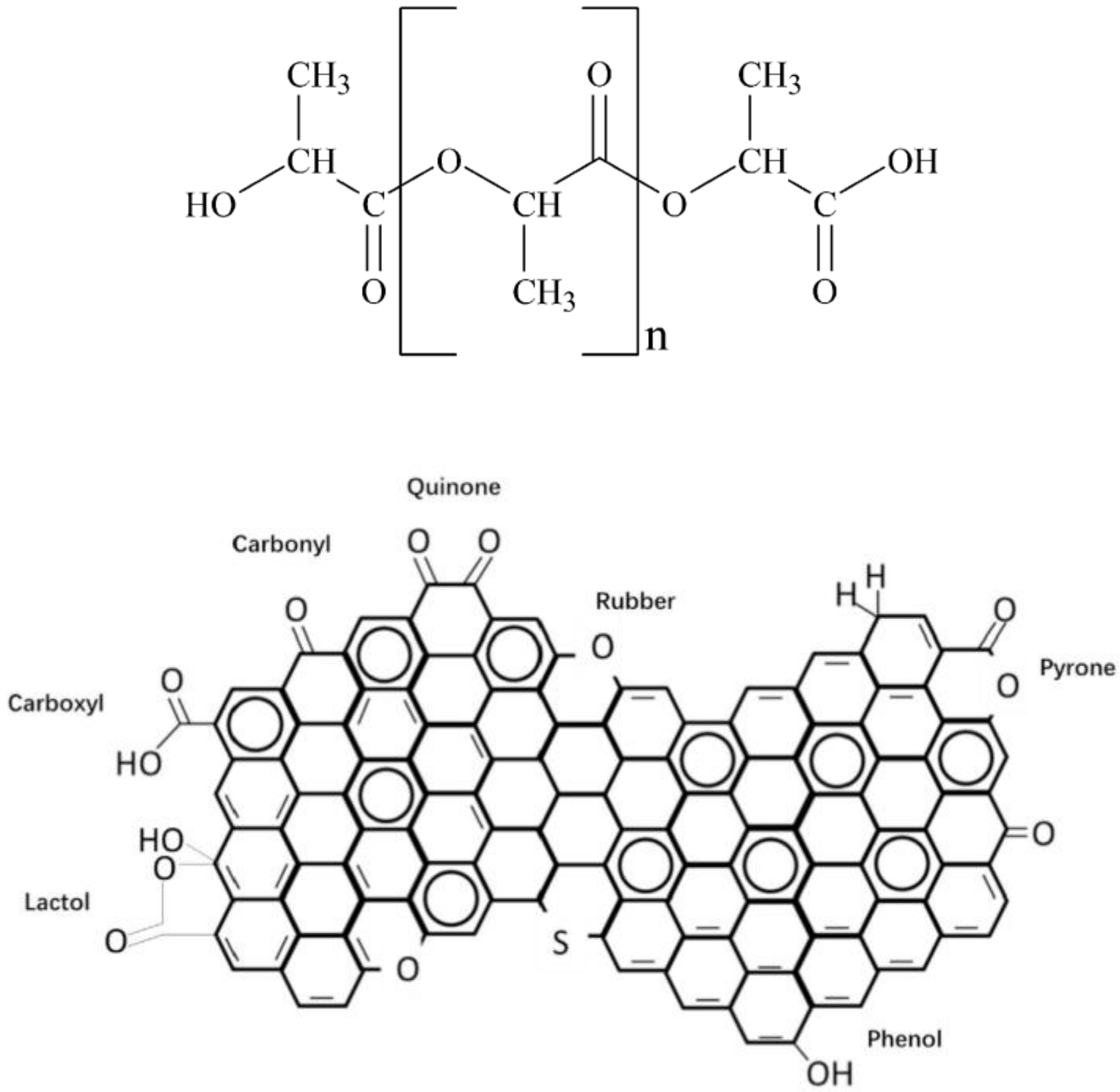

Figure S16. (A) Structural formula of PLA. Reproduced with permission from Ref.[1], Copyright 2012, Elsevier. (B) Functional groups on the carbon black surface. Reproduced with permission from Ref.[2], Copyright 2020, Elsevier.

References

(1) McKeen, L. *Renewable Resource and Biodegradable Polymers*, 3rd ed.; Elsevier: Waltham,USA, 2012. https://doi.org/10.1016/b978-1-4557-2598-4.00012-5.

(2) Fan, Y.; Fowler, G. D.; Zhao, M. The Past, Present and Future of Carbon Black as a Rubber Reinforcing Filler – A Review. *J. Clean. Prod.* **2020**, *247*, 119115. https://doi.org/10.1016/j.jclepro.2019.119115.
